# A novel Microproteomic Approach Using Laser Capture Microdissection to Study Cellular Protrusions

**DOI:** 10.3390/ijms20051172

**Published:** 2019-03-07

**Authors:** Karine Gousset, Ana Gordon, Shravan Kumar Kannan, Joey Tovar

**Affiliations:** Biology Department, California State University Fresno, 2555 East San Ramon Ave M/S SB73, Fresno, CA 93740, USA; anagordonbc@gmail.com (A.G.); shra1kumr@mail.fresnostate.edu (S.K.K.); jtovar@rocketmail.com (J.T.)

**Keywords:** Laser Capture microdissection, microproteomics, cellular protrusions

## Abstract

Cell–cell communication is vital to multicellular organisms, and distinct types of cellular protrusions play critical roles during development, cell signaling, and the spreading of pathogens and cancer. The differences in the structure and protein composition of these different types of protrusions and their specific functions have not been elucidated due to the lack of a method for their specific isolation and analysis. In this paper, we described, for the first time, a method to specifically isolate distinct protrusion subtypes, based on their morphological structures or fluorescent markers, using laser capture microdissection (LCM). Combined with a unique fixation and protein extraction protocol, we pushed the limits of microproteomics and demonstrate that proteins from LCM-isolated protrusions can successfully and reproducibly be identified by mass spectrometry using ultra-high field Orbitrap technologies. Our method confirmed that different types of protrusions have distinct proteomes and it promises to advance the characterization and the understanding of these unique structures to shed light on their possible role in health and disease.

## 1. Introduction

Cellular protrusions are involved in numerous biological functions, including cell migration, neurite outgrowth, phagocytosis, and cell–cell communication. Many are also implicated in pathological processes such as cancer–cell invasion [1] and the intercellular transmission of misfolded proteins [2] and infectious pathogens [3]. These physiological and pathological functions are controlled by different subtypes of protrusions, such as pseudopodia [1], filopodia [4], growth cones (GCs) [5,6], or tunneling nanotubes (TNTs) [2,7,8]. Interestingly, these different types of subcellular protrusions can be distinguished by light microscopy. For instance, GCs, which play an important role in axonal guidance, are specialized structures at the tip of axons, which comprise a core region with thin filopodia and lamellipodia at the periphery [5,6]. On the other hand, filopodia and TNTs are actin-rich “finger-like” structures that can only be distinguished by light microscopy from their differences in adhesion to the substratum. Indeed, filopodia, which are important sensory organelles, adhere to the substratum [9], while TNTs, which transport intercellular material [2,7,8], “hover” above the substratum [10].

Unfortunately, while some functions have been characterized, many unanswered questions about their distinct mechanisms of formation, composition or signaling pathways remain, making it difficult to reach a consensus on their nomenclature and function [11].

The first step needed to characterize their formation, function, and regulation, is to identify and compare their proteomes. Regrettably, no method currently exists to isolate disparate subtypes of protrusions. Subcellular fractionation has been used to isolate GCs [12,13]; however, the selectivity and purity of this method remains questionable, and its use with other protrusions is not possible. Other studies employ Boyden chambers [14] or excimer laser-assisted etching [15]; however, as with fractionation, these methods cannot isolate specific subsets of protrusions and are therefore limited and only appropriate for the study of protrusions in general. Thus, no technique has been described that can specifically isolate distinct subtypes of protrusions.

Here, we describe a novel method that combines Laser Capture Microdissection (LCM) and mass spectrometry (MS) to study specific subtypes of protrusions (Figure S1). LCM is a way to microscopically visualize, segregate, and enrich specific cells of interest in a heterogeneous sample for the subsequent study of their DNA, RNA, and protein without altering downstream analyses [16]. LCM has been successfully applied to isolate tumor cells [17], neurons affected by Parkinson [18] or Alzheimer disease [19], virus-infected cells [20], organelles [21], and GCs [22]. For genomic and transcriptomic studies, amplification methods exist, which lessen the need for large sample sizes. Unfortunately, for proteomic studies, no analogous amplification method currently exists. Thus, the use of LCM for proteomic studies has been limited by large sample size demands. However, recent advances in the sensitivity, detection limits, and acquisition rates of mass spectrometers based on quadrupole, ion-trap, and ultra-high field Orbitrap technologies have allowed for an unprecedented depth of analysis of low-abundance and high-complexity samples [23]—due to their high mass accuracy and specificity—giving rise to what has been termed microproteomics (i.e., the identification of proteins from complex, yet microscale samples) [23,24,25]. Using such technology, we recently described an LCM/MS method that efficiently identified the proteome of 1000 cells by improving both the fixation and protein extraction protocols [26].

In this study, we successfully pushed the limits of LCM/microproteomics to the subcellular level and compiled the proteomes of distinct subtypes of protrusions. Since the proteome [12,13] and transcriptome [22] of GCs have been characterized, we used these published studies to validate our method. Our LCM samples showed an extremely high overlap with those studies, demonstrating that microscale samples can accurately match the results of high-throughput samples, albeit at lower coverage rates depending upon the number of microscale samples analyzed. 

To further validate this method, we isolated different categories of protrusions such as axons/dendrites/GCs from serum-deprived, differentiated neuronal CADs (dCADs), identified their proteomes, and compared them to filopodia/TNTs/GCs from neuronal CADs. We showed that these subtypes of protrusions possess unique proteomes, which is underscored by differences in their functional annotations and localization. Finally, we showed that the additive—“in two sample”—approach currently used in high-throughput proteomic studies, arbitrarily eliminates critical proteins from consideration and should be used cautiously in microproteomic studies. We demonstrate that this type of analysis leads to the rejection of relevant proteins, and obscures information regarding low abundant proteins in such samples. Instead, we propose a subtractive approach that dramatically improves the identification of low abundant proteins in small samples (Figure S1). Overall, this method will significantly advance our knowledge of these specialized structures and will help to answer questions about their distinct formation and function that had thus far been out of reach.

## 2. Results

### 2.1. LCM Can Specifically Isolate and Enrich Distinct Subtypes of Protrusions

Different types of protrusions such as filopodia, axons, dendrites, GCs, and TNTs in cells can be distinguished using microscopy by looking at their unique morphologies or using fluorescence markers. LCM, which uses a finely focused UV laser to cut a region of interest (ROI), is ideal to isolate these structures. In fact, a recent study demonstrated that LCM could isolate GCs and their RNA content could be amplified and identified [22].

**Figure 1:**
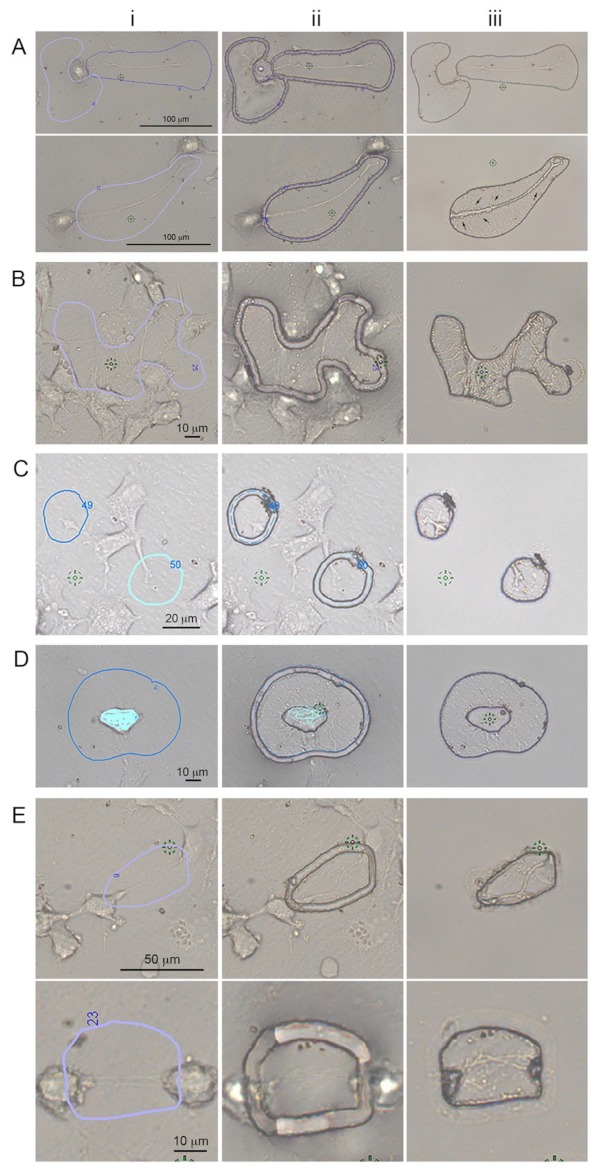
Cellular protrusions isolated by Laser Capture Microdissection. LCM, which uses a finely focused laser to cut a region of interest (ROI), is ideal to isolate cellular protrusions. Cells were plated on MMI live cell chambers, fixed, and imaged at 20× (**A**), 40× (**B**–**D**) or 60× (**E**) magnification. Various types of cellular protrusions are shown. For all cases, (i) ROIs are drawn around the protrusions of interest; (ii) are representative images of cellular protrusions after the laser cut, and (iii) images of the desired isolated cellular protrusions after removal of the LCM membrane containing the “unwanted” cells. Examples of different types of protrusions are shown: (A) axons and dendrites from dCADs are shown (scale bar = 100 μm). Small dendritic filopodia can be observed (black arrows); (B) in order to increase the number of cellular protrusions, CADs cells were treated for 5 min with 100 μM H_2_O_2_ prior to fixation. Various types of cellular protrusions are shown (scale bar = 10 μm). Individual subtypes of cellular protrusions can be specifically isolated such as (C) GCs (scale bar = 20 μm), (D) filopodia (scale bar = 10 μm), and (E) TNTs (scale bar top = 50 μm; bottom = 10 μm). TNTs do not touch the substratum and tension is visible within these structures (**E**i). As expected, after being cut, the structures collapsed onto the LCM membrane (**E**ii,iii), clearly demonstrating that these protrusions were TNTs, and not attached filopodia or other types of protrusions.

Here, we used LCM to manually select ROIs from cell cultures (Figure 1-i), cut them with the laser (Figure 1-ii), removing unwanted cells, and leaving behind only the desired ROIs (Figure 1-iii). Serum-deprived CADs (dCADs) differentiate to form axonal and dendritic protrusions [27]. These “dCAD protrusions” are large and can easily be identified and isolated by LCM (Figure 1A). Hydrogen peroxide treated CADs (hCADs) increase the number of filopodia and TNTs [28], amplifying the formation of “hCAD protrusions” such as filopodia, GCs and TNTs, which can be isolated away from the cell bodies and collected (Figure 1B). The resolution of LCM also allows for the isolation of individual subtypes of protrusions, such as GCs (Figure 1C) or filopodia (Figure 1D). TNTs, a relatively new type of cellular protrusion, are extremely fragile—and by definition do not touch the substratum [10]; however, remarkably, they could also be isolated using LCM (Figure 1E). As expected, after being cut, the structures collapsed onto the LCM membrane, clearly demonstrating that these protrusions were TNTs and not attached filopodia or other types of adhering protrusions.

### 2.2. LCM/MS Data Acquisition of Cellular Protrusions

The quality and quantity of the identified proteins obtained by MS from small fixed samples can be significantly improved by isolating samples fixed with dithiobispropionimidate (DTBP) rather than using glutaraldehyde, and using a protein extraction protocol containing Radioimmunoprecipitation assay buffer (RIPA) with dithiothreitol, 2% Sodium dodecyl sulfate, and heat [26]. We used this protocol to isolate by LCM and process for MS dCAD protrusions (Figure 1A), hCAD protrusions (Figure 1B), and GCs (Figure 1C) from CAD cells, along with each of the control whole cell samples (i.e., dCADs, hCADs and CADs). Negative controls (i.e., LCM membranes isolated around cells) were also obtained and processed for MS.

In all cases, 5 μg of proteins extracted from pooled samples of LCM-isolated cellular protrusions or 3 μg of whole cell samples were run on a limited gel and processed for LC/MS-MS. All data were collected using an Orbitrap Fusion mass spectrometer and the raw data were searched with Byonic software using a reverse-decoy strategy. Byonic data were filtered and presented at a 1% false discovery rate.

Prior to developing our improved fixation/extraction method [26], we isolated and pooled together 12,000 glutaraldehyde-fixed LCM TNT cuts (Figure 1E) [28] to determine if proteins could be identified from these fragile, transient structures. The main difference between glutaraldehyde and DTBP fixation on downstream MS analysis was not the type of proteins isolated and identified, but the quality/quantity obtained [26]. Thus, the TNTs were processed like the other subtypes of protrusions above. In total, 18 samples were obtained by LCM/MS.

**Figure 2:**
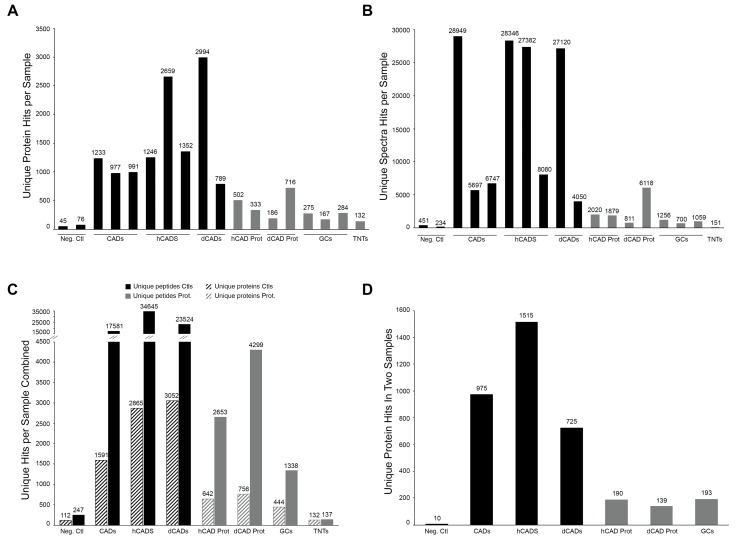
LCM/MS Microproteomic data. MS data were obtained for 18 different samples. (**A**) The number of Unique Protein or (**B**) Spectra Hits per sample are shown. (**C**) The number of unique proteins and peptide hits for all of the samples combined are represented. (**D**) A representation of the unique protein hits in at least two samples is shown. High quality/quantity of identified proteins with our DTBP-fixed samples are observed. The TNT LCM/MS data from glutaraldehyde-fixed cells resulted in mostly single peptide hits per identified proteins.

Representative graphs of the number of unique proteins for each sample are shown in Figure 2A. Variation in the number of protein hits obtained between each replicate sample did occur but appeared to be independent of the sample source. Indeed, variation in the number of unique proteins identified per sample occurred in both controls and protrusion samples. The same is true for the unique number of spectra obtained (Figure 2B). While we obtained fewer protein identifications in the cellular protrusion samples compared to controls, we were still able to get hundreds of proteins from the cellular protrusions (Figure 2C). As expected, we obtained a good quality/quantity ratio of identified proteins with our DTBP-fixed samples compared to the TNT LCM/MS data from glutaraldehyde-fixed cells. Indeed, with glutaraldehyde fixation, we only identified 132 proteins from mostly single peptide hits, compared to other protrusion subtypes that had multiple peptide hits per protein (Figure 2C). However, this does not preclude its analysis and comparison. For instance, mammalian breast cancer tissue sections microdissected by LCM were analyzed by nano-LC-ESI-MS/MS for cancer-specific biomarkers [17]. While they mostly identified proteins from single peptide hits, some were later identified to be cancer-specific biomarkers. Overall, we obtained 190, 139, and 193 unique protein hits—in two samples—for hCAD protrusions, dCAD protrusions, and GCs, respectively (Figure 2D). 

### 2.3. Reproducibility of Microproteomic Data Obtained by LCM/MS

To evaluate the reproducibility of our replicates, we employed a robust and widely used label-free spectral counting method called normalized spectral abundance factor (NSAF) [29]. NSAF determines the relative abundance of proteins in complex samples by normalizing spectral counts within samples to their respective protein length [29]. Thus, this method helps to uncover less abundant and shorter length proteins that have less available spectra to quantify. For differential expression analyses between samples, we normalized NSAF counts using DESeq [30]. Finally, since NSAF counts normalized with DESeq resulted in non-normal data, we used Johnson transformations to approximate normality for use with the Pearson correlation test. Please note that for correlation testing, we kept distinct isoforms of a single protein separate, while for all other analyses, distinct isoforms of a single protein were combined. Using this workflow, we then generated scatter plots, and their corresponding Pearson correlation, between whole cell replicate samples (Figure S2A–C) or protrusion replicates (Figure S2D–F) and between protrusions and their respective control cell samples (Figure S2G–I). A summary of all correlation values is presented in Supporting Figure S2J. We found a moderately strong correlation between GC/CADs, which may reflect the specialized functions of GCs (i.e., signal transport) compared to the neuronal cell body, and strong correlations between all other samples (Figure S2g–i). Interestingly, the average Pearson correlation coefficient for our TNT sample (*r*_avg_ = 0.648) when compared to its control whole cell CAD samples, was higher than that of other types of protrusions (Figure S2J), further validating its analysis.

From this analysis, we found that a strong correlation exists between all of these samples. Overall, these data demonstrated that LCM collected samples are reproducible, and that proteins can be identified from low abundant, microproteomic samples.

**Figure 3:**
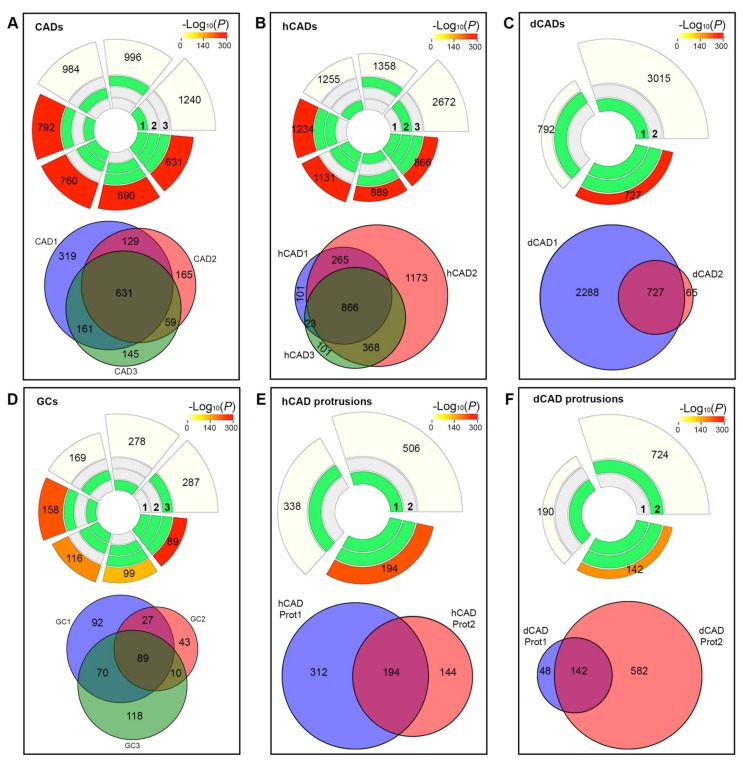
Reproducibility of LCM/MS Microproteomic data. Data were normalized for protein length using NSAF, differential expression between samples (DESeq), transformed to obtained normal data using Johnson transformations. Circular plots (top), obtained using SuperExactTest, show the intersections and the corresponding statistics of the significant proteins identified by MS from (**A**) CADs, (**B**) hCADs, (**C**) dCADs, (**D**) GCs, (**E**) hCAD protrusions, or (**F**) dCAD protrusions. The tracks in the middle represent each protein set from individual replicate (1–3) and the green and white blocks show the “presence” or “absence”, respectively, of the proteins in each intersection. In the outer layer, the height of the bars is proportional to the intersection sizes and the number of proteins is indicated on the top of the bars. The color intensity of the bars is a representation of the significance of the intersections (−log_10_(*P*)). Highly significant intersections were observed for controls and protrusions, demonstrating that the small sample size of the protrusions doesn’t affect the accuracy of protein identification. Venn diagrams (bottom) of protein hits identified for all samples (**A**–**F**) show a strong protein overlap for all samples, further demonstrating that data analysis are not affected by sample size and that microproteomics data from LCM/MS are reproducible.

Next, we analyzed the overlaps of our samples and their corresponding enrichment and significance using the SuperExactTest R software package [31]. We plotted circular plots to visualize the intersections and the corresponding statistics of the significant proteins identified by MS from our control CAD, hCAD, and dCAD samples, respectively (Figure 3A–C). The tracks in the middle represent each protein set and the green and white blocks show the “presence” or “absence”, respectively, of the proteins in each intersection. In the outer layer, the height of the bars is proportional to the intersection sizes and the number of proteins is indicated on the top of the bars. The color intensity of the bars is a representation of the P value significance of the intersections. 

We observed a highly significant overlap between control samples. Out of the 1609 unique proteins hits in CADs, 631 (39.2%) were found in all three samples (Fold Enrichment over expected (FE) = 278; *p* val < 1.0 × 10^−307^) and 980 (60.9%) were found in at least two samples (FE_avg_ = 15; combined *p* val < 1.0 × 10^−307^) (Figure 3A-top). Similarly, in hCADs, out of 2897 protein hits, 866 (29.9%) were found in all three samples (FE = 102; *p* val < 1.0 × 10^−307^) and 1522 (52.5%) were found in at least two samples (FE_avg_ = 10; combined *p* val < 1.0 × 10^−307^) (Figure 3B-top). Next, we compared duplicate dCAD samples and found that out of 3052 protein hits, 727 (23.8%) were found in both samples (FE = 7; *p* val < 1.0 × 10^−307^) (Figure 3C-top). 

These types of analyses can be misleading, suggesting that dCADs had a much smaller overlap compared to the other two samples. However, what is missing with these plots is a visualization of the overlap of the replicate samples compared to the total proteins identified. Thus, we plotted Venn diagrams to compare the protein overlap within these samples (Figure 3A–C-bottom). These plots clearly show a high overlap between all replicates, which demonstrated that the lower % found in dCADs in two samples (Figure 3C) was not due to a lower overlap between the duplicate samples, but was due to the difference in the number of proteins identified for each trial (i.e., 3052 versus 727 proteins). In fact, out of the 727 proteins, only 65 (8.9%) were not found in the other trial (Figure 3C-bottom). Overall, the tight grouping displayed in the corresponding Venn diagrams (Figure 3A–C-bottom) visually illustrates the high overlap between the control replicates.

Remarkably, the various types of isolated protrusions displayed a similarly significant overlap. Indeed, for GC triplicates (including distinct isoforms), out of 449 proteins hits, 89 (20%) were found in all three samples (FE = 3537; *p* val = 3.50 × 10^−305^) and 196 (44.1%) were found in at least two samples (FE_avg_ = 3; combined *p* val < 1.0 × 10^−307^) (Figure 3D-top). For hCAD protrusion duplicates, out of 650 protein hits, 194 (29.8%) (FE = 26; *p* val = 3.44 × 10^−243^) were found in both samples (Figure 3E-top), while for dCAD protrusion duplicates out of 772 protein hits, 142 (18.4%) (FE = 24; *p* val = 1.00 × 10^−175^) were found in both samples (Figure 3F-top). The similarity between these samples suggests that the small sample size of the LCM-isolated protrusions is not a hindrance to the accuracy of the corresponding protein identification. Finally, Venn diagrams from each distinct subtype of protrusions (Figure 3D–F-bottom) gave a high degree of protein overlap, suggesting that data acquisition and analysis were not affected by sample size variation.

### 2.4. LCM/MS Validation Using GCs

Since we demonstrated that our LCM/MS method was reproducible and sensitive enough for protein identification from LCM isolated protrusions, we decided to further validate our method using our GC samples. GCs form at the tips of neuronal axons/dendrites and play a critical role in the formation of neuronal networks and guidance [5,6]. Three recent studies have looked at the protein [12,13] and RNA content [22] of GCs, allowing us to directly analyze and compare the proteome of our LCM/MS isolated GCs to these published studies.

**Figure 4:**
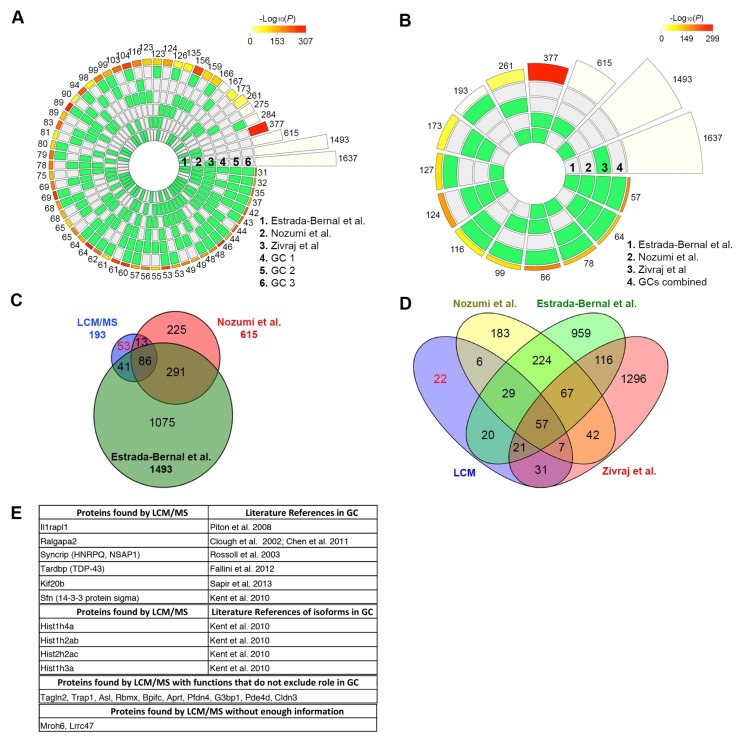
Mass spectrometry analysis of isolated growth cones. GCs from CADs were isolated by LCM and analyzed by MS. Circular plots showing the intersections and the corresponding statistics of the significant proteins identified by MS from (**A**) three independent LCM isolated GC samples (GC 1–3) or (**B**) the combined triplicate GCs compared to the proteins from proteomics [12,13] and transcriptomics studies [22] are shown. Overall, the GC proteins identified by LCM/MS matched very well with prior published data, suggesting that our isolation method is precise and that our MS analysis with a small sample is accurate. Venn diagrams of the 193 significant proteins, found in at least two of the triplicate GC samples are shown compared to (**C**) proteins identified from proteomics studies [12,13] or (**D**) proteomics [12,13] and transcriptomics [22] studies. High degrees of overlap are observed with only 53 proteins not identified in previous proteomics studies and only 22 proteins when combined with transcriptomics data. (**E**) Computation of the literature searches of the 22 proteins identified by LCM/MS, but not found in any of the other three studies, were grouped as (1) proteins found in the literature in GCs [32,33,34,35,36,37,38]; (2) with isoforms found in GCs; (3) proteins whose functions do not preclude a role in GCs; or (4) proteins without enough info. Overall, 93.8% of the proteins we identified were associated with GCs and 5.2% had functions consistent with GCs, further validating our LCM/MS method.

Thus, to validate whether our microproteomic method could accurately reproduce the results of those high-throughput studies, we compared the published proteome [12,13] and transcriptome [22] of GCs to each of our triplicate LCM/MS GC samples (combining distinct isoforms) and used circular plots to visualize the intersections and the corresponding statistics of the different protein sets (Figure 4A). Out of the 444 unique GC protein hits, 31 were found in all three high-throughput studies plus our triplicate GCs (FE = 1.05 × 10^7^; *p* val = 2.02 × 10^−209^). Additionally, 35 (FE = 7.65 × 10^5^; *p* val = 9.00 × 10^−197^), 42 (FE = 3.78 × 10^5^; p val =1.17 × 10^−223^), and 46 (FE = 1.10 × 10^6^; *p* val = 2.70 × 10^−267^) proteins were found in our triplicate GCs and at least two of the high-throughput studies and 53 (FE = 8.20 × 10^4^; *p* val = 6.30 × 10^−249^), 60 (FE = 3.49 × 10^4^; *p* val = 4.38 × 10^−260^), and 64 (FE = 4.08 × 10^4^; *p* val = 1.19 × 10^−282^) proteins were found in our triplicate GCs and at least one other high-throughput study. The extremely low *p*-values and high fold enrichment over what would be expected indicates that the overlap observed between our samples and the published studies [12,13,22] is highly significant—and not due to chance—demonstrating that LCM is both specific and reproducible.

Most MS studies, including those listed above, use an additive approach to identify important proteins, namely proteins are only considered valid hits when they are found in at least two samples. Using this approach, out of 444 proteins identified in GCs, 193 were found in at least two samples. A graphical representation of those 193 proteins compared to the published, high-throughput studies displays an even stronger overlap, with 57 (FE = 2437; *p* val = 2.77 × 10^−176^) proteins found in all four studies (Figure 4B). Moreover, 64 (FE = 176; *p* val = 3.42 × 10^−125^), 78 (FE = 78; *p* val = 8.58 × 10^−130^), and 86 (FE = 260; *p* val = 5.73 × 10^−186^) proteins were found in our GCs and at least two of the high-throughput studies. Finally, 99 (FE = 19; *p* val = 2.46 × 10^−104^), 116 (FE = 8; *p* val = 5.42 × 10^−83^), and 127 (FE = 10; *p* val = 2.79 × 10^−103^) proteins were found in our samples and at least one high-throughput study. Overall, our LCM/MS microproteomic data strongly correlate with the published, high-throughput GC data suggesting that while overall coverage may be lower, our method is both specific and accurate and can be relied upon where only microscale samples can be reasonably gathered.

A Venn diagram of the 193 identified proteins compared to the published proteomic studies further demonstrates that our LCM samples were targeted with 140 (72.5%) of our proteins found in these published studies (Figure 4C). Interestingly, by including the data from the transcriptome of GCs [22], only 22 proteins (11.4%) were found exclusively in our study (Figure 4D).

The fact that these 22 proteins were not found in the other studies does not preclude their localization within GCs. Indeed, a literature search demonstrated that six were identified in GCs, four had isoforms found in GCs, and 10 had functions that did not exclude them from GCs (Figure 4E). Only two proteins did not have enough information to explain their presence in GCs (Figure 4E). Thus, 93.8% of the proteins we identified by LCM/MS were associated with GCs, 5.2% had functions consistent with GCs, and only 1% did not have enough data to determine their GC function or localization. These results are a testimony to the accuracy of our method and demonstrate that the isolation of protrusions is not only feasible but specific and accurate.

### 2.5. Validation Using Microscopy and The Human Protein Atlas Subcell

To further validate the protein hits obtained by LCM/MS from different subtypes of cellular protrusions, we performed immunofluorescent (IF) experiments. The idea was to determine if proteins identified in specific types of protrusions by LCM/MS were in fact present in these structures.

**Figure 5:**
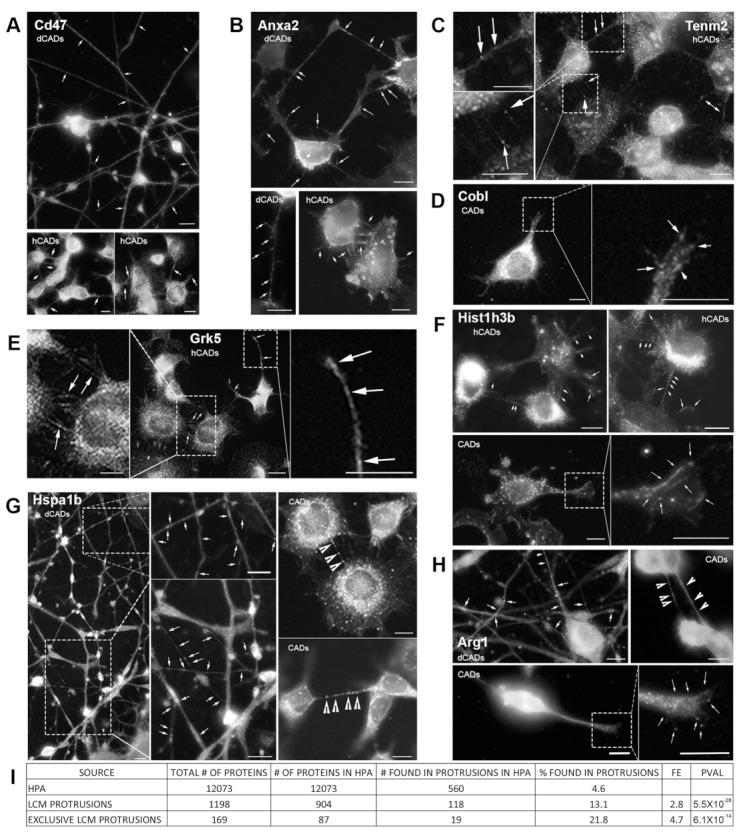
Validation of the LCM/MS data from cellular protrusions using microscopy. Immunofluorescence (IF) of proteins identified by LCM/MS (**A**–**D**) “expected” or (**E**–**H**) “not expected” to be within protrusions based on their known functions/localizations. (**A**) Cd47 and (**B**) Anxa2 are found in both hCAD/dCAD protrusions. (**C**) Tenm2 is found within hCAD protrusions and (**D**) Cobl in GCs. Interestingly, (**E**) Grk5 was observed in hCAD protrusions; (**F**) Hist1h3b protein in hCAD protrusions and GCs (**G**) Hspa1b in dCAD protrusions and TNTs and (**H**) Arg1 in dCAD protrusions, GCs, and TNTs. White arrows/arrowheads show punctates within dCAD/hCAD protrusions and GCs or within TNTs, respectively. Scale bars = 10 μm. IF of all 8 proteins corroborate the LCM/MS protein identification for each subtypes of protrusions. (**I**) 1198 unique proteins were identified by LCM/MS to be in protrusions and 169 were found to be exclusive to protrusions. 904 of the 1198 proteins found in protrusions and 87 out of 169 exclusive proteins had images in the HPA Subcell database. IF images for the proteins identified by LCM/MS in protrusions, present in the HPA Subcell database, were observed and catalogued as being observed or not in protrusions (Figure S2). On average 4.7% of proteins are found in protrusions in the HPA database, 13.6% are found in our LCM isolated protrusions and 23% were found in the “exclusive” LCM protrusions. The fold enrichment increases by 2.9% or 4.8% when we looked at proteins identified by LCM/MS in protrusions or exclusive to protrusions, respectively.

First, we looked at four proteins, whose known function and/or localization in cells could explain their presence in protrusions. The four “expected” proteins we chose were Cd47, Anxa2, Tenm2, and Cobl. In our LCM/MS data, Cd47 and Anxa2 were identified in hCAD/dCAD protrusions, while Tenm2 was found only in hCAD protrusions. IF of Cd47 showed localization throughout axons/dendrites in dCADs and in hCAD protrusions (Figure 5A). Similarly, Anxa2 was found in axons/dendrites and at the tips of dendritic filopodia in dCADs and in hCAD protrusions (Figure 5B). Numerous punctates of Tenm2 were found within hCAD protrusions (Figure 5C). Finally, Cobl, identified by LCM/MS within GCs was also observed within these structures by IF (Figure 5D).

Next, we looked at four proteins found by LCM/MS in protrusions, which we did not expect to be in protrusions based on their known function and/or localization in cells. Grk5 was identified in hCAD protrusions, while Hist1h3b was identified in hCAD protrusions and GCs. Numerous punctates of both Grk5 and Hist1h3b were found throughout hCAD protrusions, and Hist1h3b was also found in GCs (Figure 5E,F). Another unexpected protein, Hspa1b, was identified by LCM/MS in dCAD protrusions and TNTs. By IF, Hspa1b was observed throughout axons/dendrites in dCADs, and bright punctates were also observed within TNTs (Figure 5G). Finally, Arg1, identified in dCAD protrusions, GCs, and TNTs by LCM/MS was seen within these structures using IF (Figure 5H). 

Overall, IF experiments of all eight proteins confirmed their presence within the isolated protrusions identified by LCM/MS.

While our IF data corroborated our LCM/MS data, it remains anecdotal, since we only analyzed eight proteins. Since it is not feasible to individually look at all of our protein hits by IF, we took advantage of the Human Protein Atlas (HPA) Subcell database [39,40] to determine the localization of the unique proteins identified in our LCM/MS samples.

Thus, we analyzed all 12,073 proteins with images in the Human Protein Atlas (HPA) Subcell database for their localization to protrusions (Figure S3) and created a publicly accessible database—PROTPR—of the results (https://goussetlab.shinyapps.io/PROTPR/) [41]. In total, we found 560 proteins with a clear localization to protrusions (4.6%). In contrast, of the 904 proteins identified in our dCAD/hCAD/TNT protrusion samples and available in the HPA database, 118 were clearly identifiable in protrusions in the HPA (13.1%; FE = 2.8; *p* val = 5.54 × 10^−28^). Strikingly, if we only consider proteins that were exclusive to individual protrusion subtypes, 21.8% (19 out of 87) were localized to protrusions in HPA images (FE = 4.7; *p* val = 6.05 × 10^−14^) (Figure 5I). This demonstrates that our LCM isolation method significantly enriches protrusion-localized proteins. 

### 2.6. LCM/MS Data Analysis Using Annotations

Traditionally, in high-throughput proteomic studies, an additive approach is used, whereby only proteins identified in at least two samples are considered valid hits. This approach quickly eliminates unwanted proteins when working with an overwhelming number of identified proteins. However, for microproteomic studies, this approach might suppress the less abundant but functional proteins of interest. As described in Figure 2, one of the key difference between replicate samples for all types of protrusions is often the variation in the number of protein hits and not the overlap. If we take the example of dCAD protrusions (Figure 3F), out of 772 protein hits, only 142 (18.4%) would be considered valid hits using an additive approach. Since many of the proteins excluded by the additive approach were found exclusively in distinct protrusion subtypes, we wondered whether a subtractive approach [42] would increase the chances of identifying functional proteins. Indeed, subtracting common, highly abundant cytosolic, cargo, or ribosomal proteins found in other samples, should leave us with a list of lower abundant, protrusion specific, functional proteins. Thus, we re-analyzed the data and created distinct lists of proteins per sample using three different approaches: total, additive, or subtractive (Figure S4). The subtractive approach collected proteins that were found exclusively in one sample and not found in any of the other seven samples analyzed (see Figure S1, “Data Comparison”) (i.e., CADs, hCADs, dCAD, hCADs protrusions, dCAD protrusions, GCs, and TNTs).

**Figure 6:**
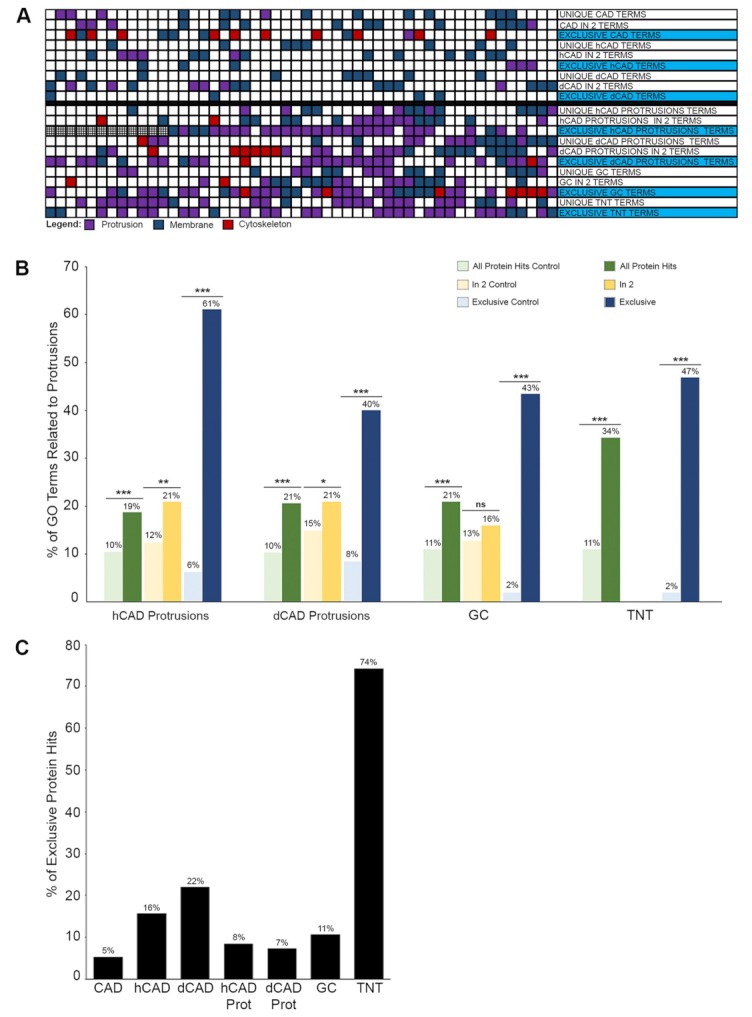
Graph analysis of cellular protrusions using annotations. (**A**) A global colorimetric view of the top 50 GO Terms for each sample highlights the increase in GO Terms related to protrusions (i.e., violet) in protrusion samples (bottom) compared to whole cells (top), confirming the HPA data analysis (Figure 5I). (**B**) Comparison of the % of GO Terms related to protrusions based on additive (proteins in 2 samples) or subtractive (exclusive to one type of protrusion) analyses, with a *p* value < 0.001 (***), < 0.01 (**), or < 0.05 (*). Additive analysis (yellow) on small LCM/MS protrusion samples is not optimal compared to the subtractive approach (blue), which resulted in 40–60% GO Terms enrichment. By subtracting the most abundant proteins found, thereby creating a list of “exclusive proteins”, less abundant, most significant proteins were identified in all subtype of protrusions. (**C**) Graphical representation of the % of exclusive protein hits per type of protrusions show a distinct proteome for each protrusion subtypes.

To examine the effect of this type of analysis, we obtained the GO Terms lists for all the samples and compared the differences between the three types of approaches (Figure S5A–G). What became apparent with these different approaches is that the effects were more pronounced with the subtypes of protrusions. A global colorimetric view of the top 50 GO Terms for each sample highlight the increase in GO Terms related to protrusions (i.e., violet) in protrusion samples (bottom) compared to whole cells (top) (Figure 6A), confirming the HPA data analysis (Figure 5I). Overall, using GO Term Enrichment analyses, we demonstrated that the subtractive approach yields lists of proteins that are exponentially more related to protrusions than using either total protein hits or the additive approach (Figure S5).

To better characterize the difference in analysis methods, the percentage of GO Term related to protrusions (Figure S5H) was plotted for all four subtypes of protrusions, for each different approach (Total = “unique protein hits;” Additive = “in 2 samples;” and Subtractive = “exclusive” protein hits) (Figure 6B). As this graph demonstrates, a large amount of data are lost when using the additive approach (Figure 6B-yellow bars) since it reduces the difference between the protrusion samples and their respective whole cell controls. In comparison, by using the subtractive approach (Figure 6B-blue bars), the percentage of GO Terms related to protrusions increased by 10-fold in hCAD protrusions, five-fold in dCAD protrusions, 21.5-fold in GCs and 23.5-fold in TNTs (Figure 6B). Thus, by using the subtractive approach, we can identify proteins that are less abundant but more related to protrusions.

A graph of the percentage of exclusive protein hits found per sample demonstrates that each of the protrusion types analyzed contain a set of proteins that is unique (Figure 6C). For example, TNTs were found to have the most unique sets of proteins with 74% of protein hits found to be exclusive to TNTs. The differences obtained between each group and the uniqueness observed with TNTs further demonstrated the specificity of our isolation method and for the first time showed that a clear distinction exists between subtypes of protrusions at the protein level.

**Figure 7:**
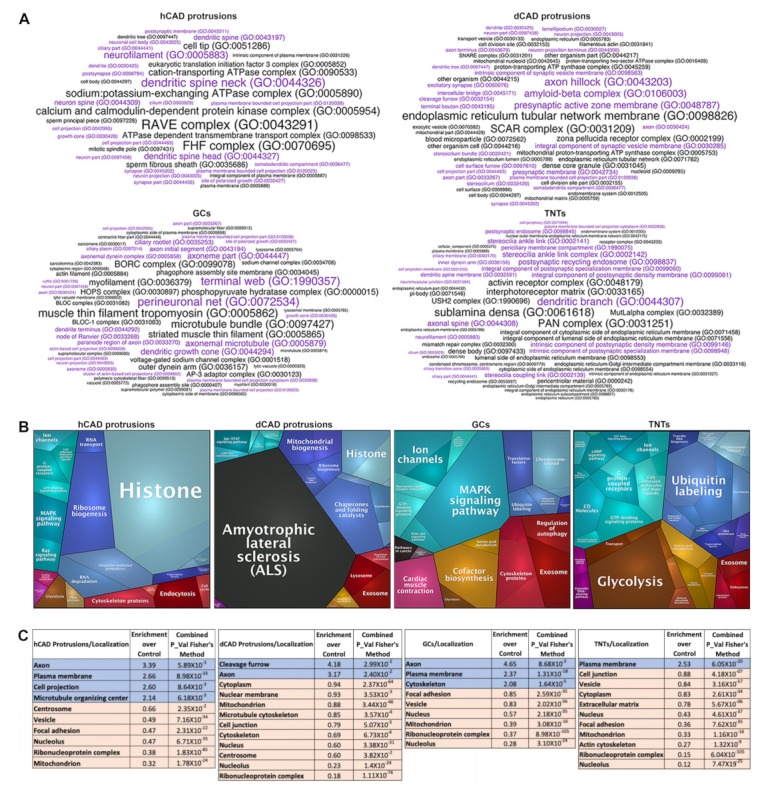
Microproteomics Analysis of cellular protrusions using annotations. (**A**) Functional categories from Gene Ontology enrichment analyses (FIDEA Enrichment word clouds) using the subtractive approach are shown for hCAD protrusions, dCAD protrusions, GCs and TNTs. GO Terms related to protrusions (Figure S5H) are shown in violet. A clear enrichment of GO terms related to protrusions is observed for all 4 subtypes. (**B**) Proteomaps for all 4 subtypes show striking differences. Major annotations for GCs and TNTs are corroborated by published studies [43,44,45,46,47,48,49], further validating our LCM/MS approach. (**C**) COMPleat (COMPutational Localization Enrichment Analysis Tool) analyses showing the subcellular localization enrichment of the 4 subtypes of protrusions are shown. The blue and orange values represent localizations that were found to be either enriched or reduced within each individual sample compared to their controls. Overall, both functional and localization analyses highlight clear differences between the cellular protrusions analyzed.

Next, we used Functional Interpretation of Differential Expression Analysis (FIDEA) Enrichment word clouds to look at cellular component functional annotations for all samples using both the additive and subtractive approaches (i.e., proteins found “in 2” samples and compared them to proteins “exclusive” to each sample) (Figure S6A,B). As expected, very specific and distinct word clouds were obtained for each subtype of protrusions. The overall enrichment of GO Terms related to protrusions (violet) in the subsets of isolated protrusions is substantially increased when using the subtractive approach (Figure S6A,B) and differs between protrusion subtypes, such as hCAD/dCAD protrusions, GCs, or TNTs (Figure 7A).

Next, we compared the Proteomaps of CAD control (Figure S6C, i-top) versus GCs (Figure S6C, ii-top), hCAD control (Figure S6C, i-center) versus hCAD protrusions (Figure S6C, ii-center), or dCAD control (Figure S6C, i-bottom) versus dCAD protrusions (Figure S6C, ii-bottom) using the additive (“in 2”) or subtractive (“exclusive”) approaches, in order to highlight differences between samples. Once again, the changes between the controls and protrusions were minimal with the additive approach (Figure S6, i versus ii “in 2”), while the Proteomaps using the subtractive approach were drastically different (Figure S6, i versus ii “exclusive”). The uniqueness in the Proteomaps obtained for hCAD protrusions, dCAD protrusions, GCs, and TNTs are highlighted in Figure 7B.

Finally, to analyze our data for its subcellular localization enrichment, we created a tool, in R, called COMPleat (COMPutational Localization Enrichment Analysis Tool) which utilizes the HPA and COMPARTMENTS localization databases. Similar to what we found using FIDEA Enrichment word clouds and Proteomaps, an analysis of the subcellular localization enrichment display clear differences between the various subsets of cellular protrusions, highlighting their uniquness.

## 3. Discussion

Cellular protrusions play a significant role in multicellular processes and have distinct functions. To date, it has not been possible to isolate and separate these various structures, which has hindered research regarding their protein composition, formation, and function, as well as the identification of biomarkers for their characterization in tissue samples or in vivo. Here, we described a novel method that allows for the specific isolation of individual protrusions, based on morphology. Using LCM/MS, we identified the proteome of GCs, which overlapped strongly with published data [12,13,22].

In order to further validate our LCM/MS method, we identify four “expected proteins” that were known to be associated with membranes and/or cellular protrusions and four “unexpected proteins” based on their known function and/or cellular localization (Figure 5). In our LCM/MS data, the membrane protein Cd47, known to localize and induce filopodia formation [50], and the phospholipid-binding protein Anxa2 [51] were identified in hCAD/dCAD protrusions. IF experiments showed that both proteins localize throughout axons/dendrites in dCADs and in hCAD protrusions (Figure 5A). In addition, Tenm2, another protein shown to induce filopodia formation and to localize to filopodia [43], was found by LCM-MS in hCAD protrusions. In agreement, numerous punctates of Tenm2 were found within hCAD protrusions by IF (Figure 5C). Finally, the actin nucleator Cobl identified by LCM/MS within GCs was observed within these structures by IF (Figure 5D), in agreement with recent findings [44].

Next, to specifically determine the purity of the LCM isolated protrusions, we analyzed the proteomes of the different subtypes of cellular protrusions, to specifically look for proteins that would appear “out of place” based on their known functions and/or cellular localization. We decided to look by IF, whether or not these proteins could be found within the cellular protrusions from which they were isolated and identified by LCM/MS, or whether this might be from cell contamination. The four “unexpected” proteins we looked at were Grk5, which regulates microtubule nucleation and normal cell cycle progression [45], the nuclear histone protein, Hist1h3b, the molecular chaperone Hspa1b, and Arg1, a urea cycle enzyme essential for mouse embryonic development [46]. As shown, in Figure 5E–H, the presence of all four “unexpected” proteins was confirmed by IF within the isolated protrusions identified by LCM/MS. Overall, these IF experiments further demonstrate the purity of our LCM isolation method and demonstrate that this method can be used to identify new candidate protein in specific subtypes of cellular protrusions.

To further demonstrate that our LCM isolation method significantly enriches protrusion-localized proteins, we used the Human Protein Atlas (HPA) Subcell database [39,40]. The fact that HPA images are limited to only 12,073 proteins, with just a few images per antibody, and that those images are not optimized for the visualization of protrusions, suggests that this HPA enrichment analysis likely understates the actual enrichment. In fact, of the eight proteins that we tested, only six were in the HPA database, and of those, merely four could be clearly identified in protrusions (Figure S3B). Thus, anecdotally, this enrichment may be significantly understated, possibly by as high as 33%.

Next, we turned our attention to the current methods of proteomics analysis. We demonstrated that approaches used in high-throughput proteomics studies, where large datasets are acquired, were not appropriate to analyze extremely small samples such as cellular protrusions. The goals of proteomics and microproteomics studies are often opposed. Most high-throughput proteomics studies start with large datasets and try to reduce the number of protein hits by looking at the proteins identified in multiple replicates. In these studies, the additive approach helps to identify the most abundant proteins. However, when looking at microproteomic datasets, such as cellular protrusions, uncovering common, yet highly abundant proteins may not yield a list of functional proteins. In fact, many common, highly abundant proteins may be found in such structures due to random Brownian motion from the cytosol, as part of the transport/cargo machinery, or as part of the local protein synthesis machinery. As such, by eliminating the highly abundant, common proteins found in whole cell samples and other protrusions, we were able to identify proteins that were found exclusively in each protrusion subtype which our analysis suggested yields a far more interesting list of proteins.

For instance, since GCs are well characterized, we further analyzed their Proteomap (Figure 7B). The most prominent annotation is “MAPK Signaling pathway.” Interestingly, this pathway plays an important role in GC regulation and navigation [47]. Another major annotation is “Ion Channels.” Again, sodium/potassium channels are present in GCs [48], along with calcium channels, which help regulate GC motility and axonal guidance [49]. “Cofactor biosynthesis/regulation of autophagy/translation factors” are other important annotations, corroborated by the know functions of GCs. Indeed, transcriptional factors and local translation in GCs have been studied [52] and autophagosome biogenesis in GCs has been observed [53]. Thus, these Proteomap annotations from the “exclusive” GC list were remarkably accurate. 

Furthermore, it is interesting to note that TNTs, whose primary function is to transport materials from one cell to another [54]—including electrical signals [55]—is the only Proteomap with “transport” as an annotation. It also contains “ion channels/cell adhesion molecules and their ligands/GTP binding signaling proteins,” all of which are consistent with TNT formation and function [11]. This demonstrates that GCs and TNTs have a distinct set of proteins with specialized functions and attests to the necessity of changing from the additive to the subtractive approach. Indeed, this method of analysis is best suited for microproteomic studies from microscale sample sizes.

Similarly, using COMPleat, we found that all protrusions were depleted in “nucleolus/nuclear" localization (Figure 7C). As expected, we observed an enrichment of “axon/plasma membrane/cell projection" localization in hCAD protrusions; “axon” localization in dCAD protrusions; “axon/plasma membrane/cytoskeleton” localization in GCs; and “plasma membrane” localization in TNTs (Figure 7C). Once again, the fact that “axon” localization was not present in TNTs is a testimony to the accuracy of our method, and the fact that we can identify and isolate small cellular protrusions amongst larger protrusions.

In addition, since the isolation of small protrusions such as TNTs can be tedious, and technical replicates are not feasible, we wanted to test whether data could be obtained from a single experiment. Interestingly, we demonstrate that this method can be used with a single dataset—in addition to multiple control samples—as an initial screening method to identify protein targets. Such protein hits can then be independently confirmed and categorized using microscopy, biochemical experiments, or functional annotations (Figure 5 and Figure 7).

Overall, using LCM/MS, we also demonstrated that the proteomes of different subtypes of protrusions are unique and corroborate with their proposed functional roles (Figure 6 and Figure 7). Finally, using this approach, we identified proteins never determined to be part of protrusions (Figure 5E–H), further highlighting the usefulness of such a method.

Up to now, researchers interested in specific subtypes of protrusions, had to rely on candidate-based approaches. Our methodology can now directly identify proteins contained within these structures and may uncover the unique proteins involved in the formation or function of each subtypes of protrusions. 

For instance, since several cancers and diseases [11,56] appear to use TNTs for cell-to-cell transmission, there is a need to better characterize these unique structures. Interestingly, a recent paper using correlative cryo-EM has demonstrated that while filopodia and TNTs look similar by light microscopy, they are structurally different [57]. Using our LCM/MS method, we are currently in the process of gathering new TNT samples along with filopodia only samples, which should finally bring to light some of the key structural and functional differences between these two types of protrusions. The identification of TNT-specific proteins could help distinguish the different transport mechanisms involved in specific diseases and lead to specific therapeutics [58]. Beyond that, it may also lead to the discovery of specific biomarkers for in vivo studies, an important limitation that has slowed down research in the TNT field [11].

**Figure 8:**
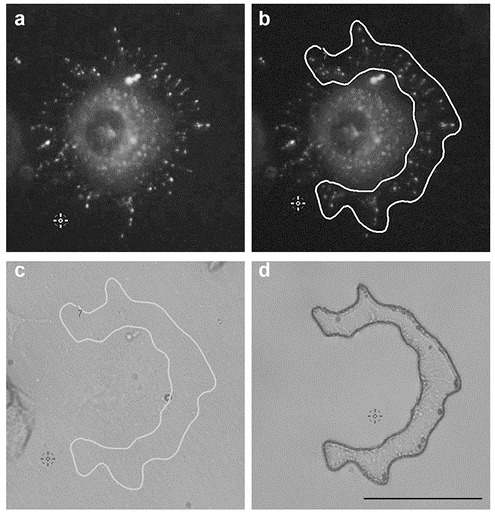
Isolation of GFP-Myo10 filopodia in transfected CADs using IF laser capture microscopy. (**a**) Representative image of a GFP-Myo10 transfected CAD cell by IF, (**b**) ROI drawn around GFP-Myo10 positive filopodia using fluorescence settings or (**c**) using the phase contrast settings. (**d**) LCM isolated filopodia after removal of unwanted cells. (Scale bar = 50 μm).

Another advantage of this approach is its versatility. Indeed, isolation with LCM is not restricted to cell morphology, as structures of interest can also be identified using fluorescence labeling. For instance, Myosin-X (Myo10) plays a role in both filopodia and TNT formation [28]. Using LCM combined with fluorescence microscopy we are currently isolating Myo10-dependent filopodia (Figure 8), to identify their key components by MS.

## 4. Materials and Methods

### 4.1. Cell Culture

Cath.A differentiated (CAD) cells, derived from a catecholaminergic neuronal tumor, were obtained from mouse B6/D2 F1 hybrid; Sigma-Aldrich (St Louis, MO, USA) under the control of the European Collection of Authenticated Cell Cultures (ECACC), which assures both the authentication of the cell line and that it is mycoplasma free) and cultured with Opti-MEM Reduced Serum Medium, GlutaMAX Supplement (Gibco Life Technologies, Carlsbad, CA, USA) and 10% fetal bovine serum (FBS; Biowest, Riverside, CA, USA). To differentiate CAD cells into neurons, cells were grown in Opti-MEM without FBS for 10 days. 

### 4.2. Fixation Protocol 

Cellular protrusions are fragile and require strong fixative solutions to preserve their structures. We have previously demonstrated that the optimal fixation conditions for immunofluorescence of TNTs, one of the most delicate type of cellular structures to preserve due to their nature (i.e., not touching the substratum), was using a fixative containing glutaraldehyde (0.05%; Sigma-Aldrich), 2% of paraformaldehyde (PFA; Electron Microscopy Sciences, Hatfield, PA, USA) and 0.2 M HEPES (Life Technology, Carlsbad, CA, USA) in phosphate-buffered saline (PBS) for 15 min, followed by a second fixative containing 4% PFA and 0.2 M HEPES in PBS for another 15 min [28]. Unfortunately, while this fixation is ideal for maintaining these structures, the MS results showed lower quantity and quality of the identified proteins, with mostly single peptide hits. For this reason, a novel fixative protocol based on dithiobispropionimidate (DTBP; ThermoFisher, Carlsbad, CA, USA), a chemical that can be de-crosslinked, was developed in order to optimize both the maintenance of the structural integrity of TNTs, as well as for the MS recovery. This new method greatly improved the quantity and quality of the proteins identified by MS [26]. Briefly, CAD cells were fixed using 4% PFA and 0.2 M HEPES in PBS for 15 min, followed by a fixative containing 5mM DTBP and 25mM HEPES in PBS for another 15 min. 

For immunofluorescence experiments where samples were not analyzed by MS, the glutaraldehyde-based protocol was used. 

### 4.3. Laser Capture Microdissection

LCM was performed using a laser microdissection system from Molecular Machines and Industries (MMI CellCut Laser Microdissection, Eching, Germany) controlled by the MMI Cell Tools Software from the same company.

#### 4.3.1. LCM Dish Preparation

The MMI Live cell chambers with membrane and petri dish (MMI, Haslett, MI, USA) were used to isolate all types of cellular protrusion by laser capture microdissection (LCM). The attachment of the cells to the membrane was facilitated by treating the membrane with 6 mM of fibronectin (Sigma-Aldrich) in PBS for 20 min at 37 °C. The solution was then removed and the membrane was washed twice with filtered PBS and once with Opti-MEM. Next, CAD cells were cultured in the dish and incubated at 37 °C for 3 h to allow the cells to attach to the surface and make protrusions. It should be taken into account that the cell density is a critical step and will depend on the cell line and the type of protrusion of interest (i.e., 60,000 or 180,000 CAD cells were plated to isolate growth cones or TNTs, respectively). In addition, cellular protrusions can be increased by different approaches, in the current work we used an exposure to 100 µM H_2_O_2_ (Fisher Scientific, Hampton, VA, USA) in Opti-MEM for 5 min [28].

#### 4.3.2. Laser Calibrations

The MMI system uses a fixed UV-laser with high pulse rate and low power, allowing for sharp and precise cutting line of 0.3 to 0.5 microns.

Laser cut speed: the laser cut speed was set low in order to avoid removing cells and/or the fragile cellular projections of interest during the cutting process. The speeds used in this study were in the range of 10 to 35 µm/s depending on the type of cellular protrusion cut. TNTs, which were the most fragile types of protrusions, were cut at 10 µm/s, while other adhering protrusions were cut at 20 or 35 µm/s, with three cutting repetitions (i.e., the laser cut the same area three times in order to ensure that the membrane and cellular protrusions were entirely cut from the rest of the dish).

Laser focus: the Laser focus (i.e., the position of the laser beam in the Z-direction) was adjusted before each experiment to ensure optimal and precise cutting. This was ensured by calibrating the laser focus according to the plane tilt of the dish. The laser focus used in this study was in the range of 2500 µm to 3400 µm.

Laser power: the power needed to samples is normally proportional to the sample thickness. The laser power was always set to get the smallest, precise laser cut through the membrane and was calibrating along with the Laser focus before each experiment. The power used in this study was in the range of 60% to 75%.

#### 4.3.3. Objective Used

To improve the enrichment of different cellular protrusion from the cells of interest, the settings for LCM were adjusted for the growth cones, hCAD protrusions or TNTs with a 40× dry objective. When protrusions of dCADs were longer than 250 µm, the 20× objective was used.

#### 4.3.5. Post-Cutting

To ensure that the LCM membranes, cellular debris, proteins from exosomal origin, secreted by the cells or from the FBS in the media did not contaminate our samples, negative controls were also isolated. Briefly, cells were plated similar to all other experiments, but instead of isolating specific types of protrusions, ROIs were obtained from “empty” regions in the LCM dishes, between groups of cells. These negative control samples were extracted and analyzed by MS, in an identical manner to all other samples. After isolating the different cell protrusions, the membrane rings were gently lifted from the microdissection chamber dishes, leaving the cut protrusions on the attached membrane. This step was carried out under the microscope at 4× magnification to check for any loss of cuts from the membrane while separating it from the microdissection chamber. The 40x magnification was used to verify that there was no contamination of cell bodies. 

In order to gather enough proteins for each subcellular protrusion types, LCM-isolated protrusions had to be pooled together from different dishes for each independent experiment. For all experiment subtypes of protrusions, and/or whole cells, samples were cut within five days post fixation. On day five, the cellular protrusions were removed from the LCM dishes, recovered by pipetting with modified RIPA buffer and stored in a centrifuge tube at −80 °C. For growth cones, ~5000–6000 cuts from three dishes were pooled together for all three independent experiments. For hCAD protrusions, ~1000 cuts were obtained from three LCM dishes and for dCAD protrusions, ~1000–2000 cuts from two LCM dishes were pooled together for both duplicate experiments and stored at −80 °C until extraction. The cuts obtained for the different subcellular were similar to the example shown in Figure 1. For each independent experiment, the growth cones and hCAD protrusions took ~2–3 weeks to isolate and ~2 weeks for the dCAD protrusions. 

In the case of whole cell samples, the complete membrane was separated from the ring and immersed in modified RIPA buffer. All samples were stored at −80 °C until protein extraction.

For TNT isolation from cells fixed with glutaraldehyde/PFA fixation (see above), ~12,000 cuts were isolated from ~15 dishes in RIPA buffer. All of the samples from the 15 dishes were pooled into a single tube and stored at −80 °C until protein extraction. It took ~3–4 months to gather enough samples for one independent MS experiment. 

### 4.4. Protein Extraction

As recently described [26], the samples in modified RIPA buffer [10 mM Tris-HCl (pH 8.0), 1mM EDTA, 0.5 mM EGTA, 1% Triton X-100, 0.1% Sodium deoxycholate, and 140mM Sodium chloride], along with 1:100 Halt protease inhibitor cocktail (ThermoFisher)] containing 2% sodium dodecyl sulfate (SDS) and 100 mM DTT (Sigma-Aldrich) were sonicated for 5 min. Next, samples were incubated on ice for 20 min, followed by incubations at 37 °C in a water bath for 30 min, at 100 °C (dry bath) for 20 min, and at 60 °C (dry bath) for 2 h. Finally, samples were again sonicated for 5 min and centrifuged for 2 min at max speed. Protein concentration was assessed using RCDC (Biorad, Hercules, CA, USA), a detergent- and reducing agent-compatible assay. 

### 4.5. Mass Spectrometry Sample Preparation

Five or three micrograms of protein lysate from pooled samples of isolated cellular protrusions or from whole cells/negative controls, respectively, were denatured using laemmli buffer containing 100 mM DTT and boiled for 5 min. Next, samples were run on 7.5% Mini-Protean TGX gels (BioRad) at 30 mAmp, for about 5–10 min, down to ~1 cm inside the gel (enough to visualize the MW marker ladder separation). This “limited gel” allows for the separation of the proteins by size in preparation for MS. The limited gel was stopped and fixed for 1h in fixing solution (Water:Methanol:Acetic acid = 40:40:8). Inside a biosafety cabinet, the samples were excised carefully from the gel and stored in 1% acetic acid water solution until analyzed by MS.

### 4.6. Mass Spectrometry—Sample Preparation for LC-MS

All mass spectrometry data collection and protein identification were contracted and performed by the Vincent Coates Foundation Mass Spectrometry Laboratory at Stanford University to eliminate bias. The analysis of the protein identification data was performed in-house. 

The samples were diced into 1 mm × 1 mm squares, rinsed multiple times with 50 mM ammonium bicarbonate and reduced with 5 mM DTT, 50 mM ammonium bicarbonate at 55 °C for 30 min. Residual solvent was removed and alkylation was performed using 10 mM propionamide in 50 mM ammonium bicarbonate for 30 min at room temperature (RT). The gel pieces were rinsed with 50% acetonitrile 50 mM ammonium bicarbonate and place in a speed vacuum for 5 min. Digestion was performed with trypsin/LysC (Promega, Madison, WI, USA) overnight digest at 37 °C. Tubes were spun and the solvent including peptides were collected, further peptide extraction was performed by the addition of 60% acetonitrile, 39.9% water, 0.1% formic acid and incubated for 10–15 min. The peptide pools were dried in a speed vacuum.

### 4.7. Experimental Design and Statistical Rationale

Digested peptide pools were reconstituted and injected onto a 100 micron I.D. C18 reversed phase analytical column (Dr. Maisch, Ammerbuch-Entringen, Germany; 2.4 μM Reprosil-Pur) 25–50 cm in length. The UPLC was a Waters M class, operated at 300 nL/min using a linear gradient from 4% mobile phase B to 35% B. Mobile phase A consisted of 0.2% formic acid, 5% DMSO and water; mobile phase B was 0.2% formic acid, 5% DMSO, acetonitrile. All data were collected using an Orbitrap Fusion mass spectrometer set to acquire data in a data dependent fashion selecting and fragmenting by collision-induced dissociation the most intense precursor ions optimized to maximize duty cycle. An exclusion window of 60 seconds was used to improve proteomic depth and multiple charge states of the same ion were not sampled. A sample of hCAD and dCAD protrusions were run on a TIMS TOF, which gave comparable results. All raw MS/MS data were analyzed using Preview and Byonic v2.6.49 (Protein Metrics) as well as custom tools for data analysis developed in MatLab at Stanford University. Peak selection was handled automatically within Byonic.

MS/MS data were searched against a UniProtKB FASTA database containing 16,972 reviewed Mus musculus entries (various dates). Propionamidation (+71.037114 @ C) was set as a fixed modification, deamination (+0.984016 @ N) and Acetylation (+42.010565 @ K) were set as a common1 modifications, Oxidation (+15.994915 @ M) was set as common2 modification, and Acetylation (+42.010565 @ Protein N-term) and Methylation (+14.01565 @ K, R) were set as rare1 modifications. Byonic was set to allow a maximum of two common modifications and one rare modification and to allow a maximum of two missed cleavages. MS/MS spectra were matched with a tolerance of 12 ppm on precursor mass and 0.4 Da on fragment mass.

Common contaminants were filtered automatically by Byonic and include TRYP_PIG, ALBU_BOVIN, ALBU_HUMAN, CASB_BOVIN, CASK_BOVIN, CAS1_BOVIN, CO3_HUMAN, HBA_HUMAN, HBB_HUMAN, K1M1_SHEEP, K2C1_HUMAN, K22E_HUMAN, K1C10_HUMAN, K1C15_SHEEP, K1C9_HUMAN, KRHB1_HUMAN, KRHB3_HUMAN, KRHB5_HUMAN, KRHB6_HUMAN, TRFE_HUMAN.

The growth cone samples and their respective controls were repeated in triplicate. Since this study sought to validate our LCM collection/enrichment method against existing methods, triplicate samples (with an “in two” exclusion criteria) were chosen to match the studies of Nozumi et al. and Estrada et al. Due to the nature of microproteomic studies, technical replicates are not possible since near full injection is necessary to increase the sensitivity and coverage of microproteomic samples.

By using Byonic, the proteome was searched with a reverse-decoy strategy and all data filtered and presented at a 1% false discovery rate. Byonic calculates a Byonic score that is an indicator of the correctness of our peptide-spectrum matches (PSM). Byonic scores reflect the absolute quality of the PSM, and have proved more useful than p-values [59]. Byonic scores range from 0 to 1000, with 300 being a good score, 400 a very good score, and scores over 500 reflecting near perfect matches. Thus, all filtered protein identification hits have an FDR rate ≤ 1%, and a Byonic score >250 or a log probability >3. 

The Raw MS/MS files and Byonic search results files have been deposited in the UCSD MassIVE repository with the dataset identifier MSV000082576 and can be accessed at ftp://massive.ucsd.edu/MSV000082576.

To analyze the MS data and the reproducibility of our replicates, we used a spectral counting method known as normalized spectral abundance factor (NSAF) [29]. We have previously used this label-free quantitation method to determine the relative abundance of proteins in 1000 whole cell samples [26]. By taking into account the fact that longer proteins have more spectra available to be identified, raw MS spectral data can be normalized. Thus, using NSAF quantification we were able to analyze the abundance of proteins caught in our various samples and plotted correlation graphs. In cases where multiple isoforms of an individual protein were identified those isoforms were treated as separate proteins for NSAF quantification and correlation analysis. Negative controls (10 proteins were found in at least two samples) served as a baseline NSAF value for all other datasets. 

In order to compare the different sample sets, DESeq [30] was used to estimate the variance mean in count data, allowing the use of a negative binomial model to test differential expression. NSAF/DESeq normalization resulted in non-normal data. In order to approximate normality and to use a parametric test such as Pearson, we used Johnson transformations.

All plots and data analysis were obtained using R and the statistical analyses were done using StatPlusPro, JMP, or SuperExactTest R software package [31].

### 4.8. Quantitative Overlap

The Quantitative overlap analyses were performed as previously described using the SuperExactTest R software package [31]. The SuperExactTest was specifically developed to visualize multi-set intersections. We used this software to compare and visualize the lists of proteins identified for each MS sample and published protein lists [12,13,22]. Circular plots show the intersections and the corresponding statistics of the different protein sets. For each lot, the middle tracks represent the protein sets analyzed and the green blocks highlight the proteins that were “present”, compared to the white or “absent” proteins in each intersection. The height of the bars in the outer layer is proportional to the intersection sizes, and the number of proteins is indicated on the top of the bars. The color intensity of the bars is a representation of the P value significance of the intersections.

### 4.9. Immunofluorescence (IF) Validation Using the Human Protein Atlas (HPA) Subcell Database

We used the IF images from the 12073 proteins in the HPA Subcell database [39,40] to analyze our MS data for its localization to protrusions. IF images for all proteins were catalogued as being observed or not in protrusions. Positive hits were counted only when they were clearly identifiable in protrusions (Figure S3). These data are compiled in a publicly accessible database, PROTPR (https://goussetlab.shinyapps.io/PROTPR/).

A 2 × 2 contingency table with odds ratio was used to calculate fold enrichment over expected. Significance was tested using two separate chi square tests (Pearson’s chi-square test and Yates’s correction for continuity).

### 4.10. Immunofluorescence (IF)

Among all the proteins obtained by MS in the protrusion samples, we identified proteins that we would “expect” or “not expect” to be found in protrusions based on their known function and/or localization. These proteins were then visualized by IF in order to determine their localization within cells and whether or not they were present within protrusions. For these IF experiments, 60,000 CAD cells were plated the day before on coverslips. The next day, cells were fixed and permeabilized with methanol (7 min at −20 °C) or Triton (0.1% for 10 min at RT), depending on primary antibody used. The next steps were: blocking with 2% BSA for 1 h, incubation with primary antibody (in blocking solution) for 1 h at RT, washes with PBS, wash with blocking solution, incubation with secondary antibody (1:400 in blocking solution) for 1 h at RT, washes with PBS, mounting on slides and sealed with Aqua-poly mount (PolySciences, Warrington, PA, USA). The primary antibodies used were: rabbit anti-Grk5 (sc-11396), rabbit anti-Cd47 (sc-25773), mouse anti-Anxa2 (sc-28385), goat anti-Hist1h3b (sc-8654), and goat anti-Tenm2 (sc-165674) from Santa Cruz Biotechnology (Dallas, TX, USA) and rabbit anti-Cobl (NBP1-89615), rabbit anti-Arg1 (NBP1-32731), and mouse anti-Hspa1b (H00003304-M02) from Novus Biologicals (Centennial, CO, USA). The secondary antibodies used were Goat anti-Rabbit IgG (H+L) Cross-Adsorbed Secondary Antibody, Alexa Fluor 546; Donkey anti-Goat IgG (H+L) Cross-Adsorbed Secondary Antibody, FITC or Rabbit anti-Mouse IgG (H+L) Cross-Adsorbed Secondary Antibody, Alexa Fluor 488 (ThermoFisher Scientific). Negative controls: cells were permeabilized with either MeOH or Triton X-100 and incubated only with secondary antibodies to ensure that under our experimental conditions; we did not have nonspecific fluorescent signals from the secondary antibodies used. For each antibody, high resolution images were acquired using a widefield inverted Leica microscope (DMI3000) controlled by Metamorph acquisition software (Molecular Devices, San Jose, CA, USA), a 63×/1.25 oil objective and a Leica DFC300 FX camera. For each condition, Z-stacks were acquired and 3D cell volume rendering was obtained. Representative pictures are shown. The analyses of the Z-stacks were obtained using the Image J software (http://rsb.info.nih.gov/ij/).

### 4.11. Myosin-X Transfection

150,000 CAD cells, plated on a 35 mm dish the day before transfection, were transiently transfected with EGFPC1-hMyosin-X plasmid [60] using Lipofectamine 2000 (ThermoFisher Scientific) in accordance with the manufacturer’s instructions. 48 h after transfection, cells were split, counted and seeded (70,000 cells) on a MMI Live cell Chamber dish and fixed 3 h later as described above. The GFP-Myo10 transfected cells were identified using the FITC filter of the LCM and subsequently, membrane structures, such as tip complex, were delimited with the FITC channel and cut with the laser.

### 4.12. Localization Enrichment Analysis

The localization of the proteins identified in our samples was analyzed using a free, in-house developed R software tool called COMPLEAT (COMPutational Localization Enrichment Analysis Tool). This software identifies the enriched/depleted localizations of lists of proteins using the COMPARTMENTS and HPA Subcellular Atlas localization databases.

A 2 × 2 contingency table with odds ratio was used to determine enrichment/depletion of localizations. The significance of the difference in background frequency versus the sample frequency was analyzed with two separate chi square tests (Pearson’s chi-square test and Yates’s correction for continuity). The chi-square value was then converted using R to a *p*-value using the command pchisq (Chi-square Value, Degrees of Freedom).

This software can be found at: https://goussetlab.shinyapps.io/compleat-app/.

## 5. Conclusions

LCM, which combines light microscopy to select individual cellular protrusions and UV-laser with high pulse rate and low power to precisely cut around them, is the only method to date able to isolate and specifically enriched distinct subtypes of cellular protrusions. The LCM/MS method, we describe here, has pushed the limits of microprotemics and demonstrates that it can specifically and reproducibly identify the proteomes of distinct protrusions. Thus, it promises to open up numerous new avenues of study. 

## Supplementary Materials

Supplementary materials can be found at https://www.mdpi.com/1422-0067/20/5/1172/s1.

## Abbreviations

CAD	Cath.A differentiated neuronal cell line
dCAD	Serum-starved differentiated CAD
DTBP	dithiobispropionimidate
GC	Growth cone
hCAD	CAD cells treated with hydrogen peroxide
LCM	Laser Capture Microdissection
TNT	Tunneling nanotube
