# Supplementary material for: A novel Microproteomic Approach Using Laser Capture Microdissection to Study Cellular Protrusions
Source: Int J Mol Sci. 2019 Mar 7;20(5):1172. doi: 10.3390/ijms20051172 (PMC6429397; doi:10.3390/ijms20051172)
Supplement: Supplementary file 1 [file ijms-20-01172-s001.zip › New-Fig S5E-s.pdf]

E

UNIQUE dCAD PROTRUSION TERMS	dCAD PROTRUSION IN 2 TERMS	EXCLUSIVE dCAD PROTRUSION TERMS
**extracellular exosome (GO:0070062)**	**myelin sheath (GO:0043209)**	endoplasmic reticulum tubular network membrane (GO:0098826)
**extracellular vesicle (GO:1903561)**	chaperonin-containing T-complex (GO:0005832)	**axon hillock (GO:0043203)**
**extracellular organelle (GO:0043230)**	chaperone complex (GO:0101031)	**SCAR complex (GO:0031209)**
**myelin sheath (GO:0043209)**	**zona pellucida receptor complex (GO:0002199)**	**amyloid-beta complex (GO:0106003)**
**vesicle (GO:0031982)**	**extracellular exosome (GO:0070062)**	**presynaptic active zone membrane (GO:0048787)**
**extracellular space (GO:0005615)**	**extracellular vesicle (GO:1903561)**	**zona pellucida receptor complex (GO:0002199)**
**extracellular region part (GO:0044421)**	**extracellular organelle (GO:0043230)**	dense core granule (GO:0031045)
**extracellular region (GO:0005576)**	endoplasmic reticulum chaperone complex (GO:0034663)	**integral component of synaptic vesicle membrane (GO:0030285)**
**focal adhesion (GO:0005925)**	**extracellular matrix (GO:0031012)**	blood microparticle (GO:0072562)
**cell-substrate adherens junction (GO:0005924)**	**extracellular region part (GO:0044421)**	mitochondrial proton-transporting ATP synthase complex (GO:0005753)
**cell-substrate junction (GO:0030055)**	**extracellular space (GO:0005615)**	**presynaptic membrane (GO:0042734)**
cytoplasmic part (GO:0044444)	**vesicle (GO:0031982)**	other organism part (GO:0044217)
cytoplasm (GO:0005737)	smooth endoplasmic reticulum (GO:0005790)	other organism cell (GO:0044216)
**adherens junction (GO:0005912)**	proteasome core complex, alpha-subunit complex (GO:0019773)	other organism (GO:0044215)
**anchoring junction (GO:0070161)**	**extracellular region (GO:0005576)**	proton-transporting ATP synthase complex (GO:0045259)
cytosol (GO:0005829)	**focal adhesion (GO:0005925)**	endoplasmic reticulum tubular network (GO:0071782)
proton-transporting two-sector ATPase complex (GO:0016469)	**cell-substrate adherens junction (GO:0005924)**	**intrinsic component of synaptic vesicle membrane (GO:0098563)**
proteasome core complex (GO:0005839)	**cell-substrate junction (GO:0030055)**	**cell surface furrow (GO:0097610)**
mitochondrion (GO:0005739)	proteasome core complex (GO:0005839)	**cleavage furrow (GO:0032154)**
**membrane-bounded organelle (GO:0043227)**	**presynaptic cytosol (GO:0099523)**	**excitatory synapse (GO:0060076)**
proteasome core complex, alpha-subunit complex (GO:0019773)	citrate lyase complex (GO:0009346)	**terminal bouton (GO:0043195)**
proton-transporting two-sector ATPase complex, catalytic domain (GO:0033178)	extrinsic component of lysosome membrane (GO:0032419)	**intercellular bridge (GO:0045171)**
organelle (GO:0043226)	**adherens junction (GO:0005912)**	**axon (GO:0030424)**
**zona pellucida receptor complex (GO:0002199)**	**anchoring junction (GO:0070161)**	**axon part (GO:0033267)**
intracellular part (GO:0044424)	mitochondrial nucleoid (GO:0042645)	**stereocilium (GO:0032420)**
proteasome complex (GO:0000502)	nucleoid (GO:0009295)	cell division site part (GO:0032155)
protein complex (GO:0043234)	**VCP-NSFL1C complex (GO:1990730)**	cell division site (GO:0032153)
intracellular (GO:0005622)	**integrin alpha1-beta1 complex (GO:0034665)**	mitochondrial nucleoid (GO:0042645)
chaperonin-containing T-complex (GO:0005832)	**integrin alpha7-beta1 complex (GO:0034677)**	nucleoid (GO:0009295)
mitochondrial proton-transporting ATP synthase complex (GO:0005753)	**integrin alpha11-beta1 complex (GO:0034681)**	SNARE complex (GO:0031201)
endopeptidase complex (GO:1905369)	**integrin alpha10-beta1 complex (GO:0034680)**	**filamentous actin (GO:0031941)**
intracellular organelle part (GO:0044446)	**integrin alpha3-beta1 complex (GO:0034667)**	proton-transporting two-sector ATPase complex (GO:0016469)
chaperone complex (GO:0101031)	lysosomal matrix (GO:1990836)	stereocilium bundle (GO:0032421)
organelle part (GO:0044422)	mitotic spindle microtubule (GO:1990498)	endoplasmic reticulum lumen (GO:0005788)
intracellular organelle (GO:0043229)	sperm mitochondrial sheath (GO:0097226)	**lamellipodium (GO:0030027)**
macromolecular complex (GO:0032991)	RNA nuclear export complex (GO:0042565)	**axon terminus (GO:0043679)**
proton-transporting ATP synthase complex (GO:0045259)	NF-kappaB complex (GO:0071159)	**exocytic vesicle (GO:0070382)**
mitochondrial part (GO:0044429)	mitochondrial ribonuclease P complex (GO:0030678)	cell body (GO:0044297)
**neuron part (GO:0097458)**	endothelial microparticle (GO:0072563)	**neuron projection terminus (GO:0044306)**
**neuron projection (GO:0043005)**	**tubulin complex (GO:0045298)**	**neuron projection (GO:0043005)**
**Arp2/3 protein complex (GO:0005885)**	mitochondrial proton-transporting ATP synthase complex, catalytic core F(1) (GO:0000275)	mitochondrial matrix (GO:0005759)
mitochondrial envelope (GO:0005740)	proton-transporting ATP synthase complex, catalytic core F(1) (GO:0045261)	endoplasmic reticulum (GO:0005783)
endoplasmic reticulum chaperone complex (GO:0034663)	lysosomal lumen (GO:0043202)	**transport vesicle (GO:0030133)**
peptidase complex (GO:1905368)	**invadopodium (GO:0071437)**	mitochondrial part (GO:0044429)
cell (GO:0005623)	**alveolar lamellar body (GO:0097208)**	**cell surface (GO:0009986)**
cell part (GO:0044464)	pore complex (GO:0046930)	**cell projection part (GO:0044463)**
organelle membrane (GO:0031090)	mitochondrial matrix (GO:0005759)	**plasma membrane bounded cell projection part (GO:0120038)**
mitochondrial inner membrane (GO:0005743)	proteasome complex (GO:0000502)	endomembrane system (GO:0012505)
cell body (GO:0044297)	endopeptidase complex (GO:1905369)	**neuron part (GO:0097458)**
mitochondrial protein complex (GO:0098798)	mitochondrial inner membrane (GO:0005743)	**somatodendritic compartment (GO:0036477)**

Figure S5
