# Supplementary material for: A novel Microproteomic Approach Using Laser Capture Microdissection to Study Cellular Protrusions
Source: Int J Mol Sci. 2019 Mar 7;20(5):1172. doi: 10.3390/ijms20051172 (PMC6429397; doi:10.3390/ijms20051172)
Supplement: Supplementary file 1 [file ijms-20-01172-s001.zip › New-Fig S6C-with-legend-s.pdf]

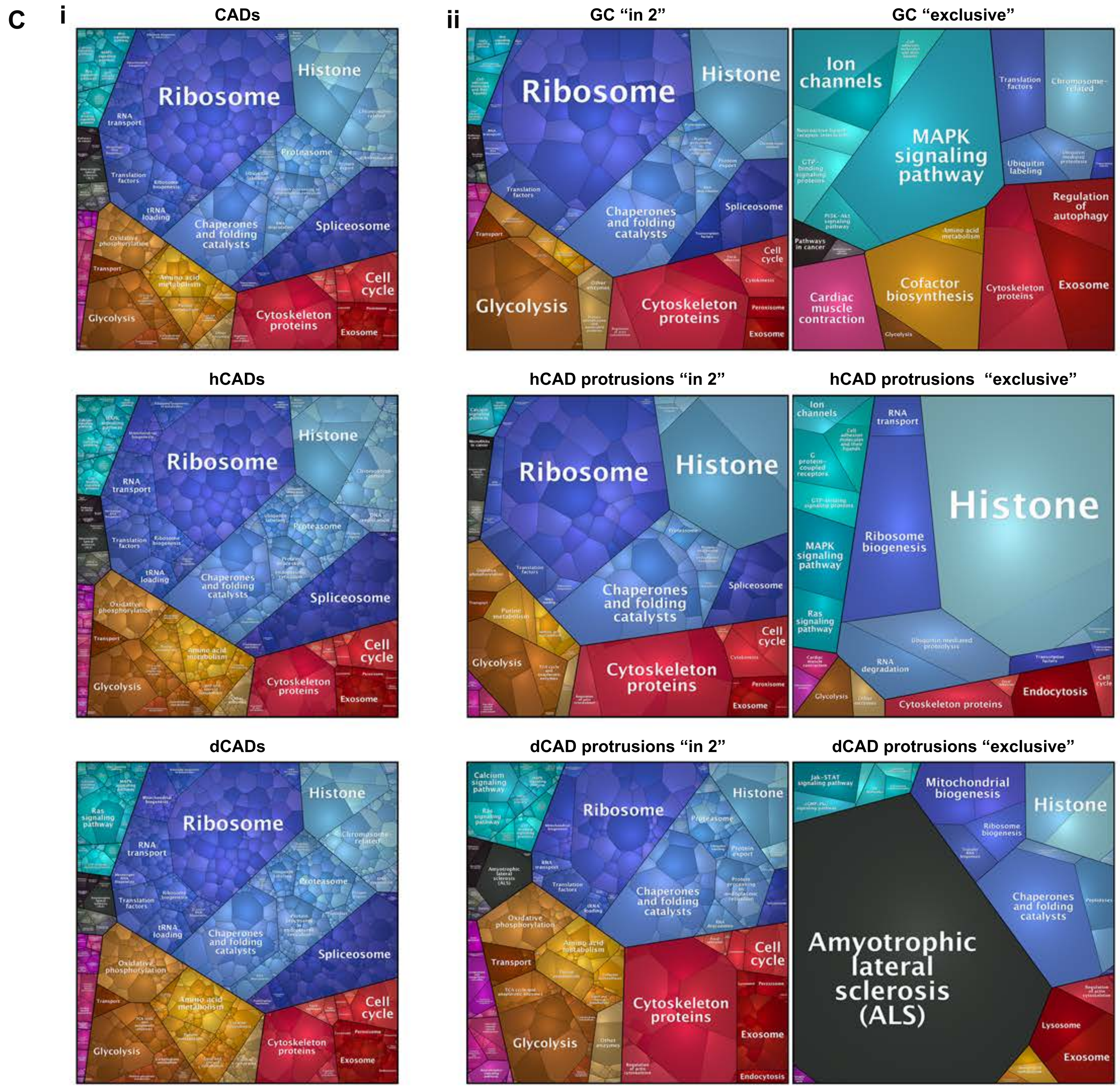


**Figure S6: Comparison of FIDEA Enrichment word clouds and Proteomaps using additive or subtractive analyses.** FIDEA Enrichment word clouds of (A) control whole cell CADs (top); hCADs (center); dCADs (bottom); or (B) hCAD protrusions (top); dCAD protrusions (center); or GCs (bottom) obtained from proteins in at least 2 samples ("in 2") (additive approach) or "exclusive" proteins to each sample (subtractive approach) are shown. (C) Proteomaps of all unique proteins from control whole cells (i) vs their individual subsets of isolated protrusions GCs (ii) ("in 2"/additive or "exclusive"/subtractive) are shown. Top: CAD (i) vs GCs (ii); Center: hCADs (i) vs hCAD protrusions (ii); Bottom: dCADs (i) vs dCAD protrusions (ii). Overall, small changes are observed between whole cell controls and their respective protrusions using the additive approach for both FIDEA enrichment word clouds and proteomaps. In comparison, drastically distinct word clouds, enriched in terms related to protrusions (ie. violet) or proteomaps are observed when using the subtractive approach.
