# Supplementary material for: A novel Microproteomic Approach Using Laser Capture Microdissection to Study Cellular Protrusions
Source: Int J Mol Sci. 2019 Mar 7;20(5):1172. doi: 10.3390/ijms20051172 (PMC6429397; doi:10.3390/ijms20051172)
Supplement: Supplementary file 1 [file ijms-20-01172-s001.zip › New-Fig S2-s.pdf]

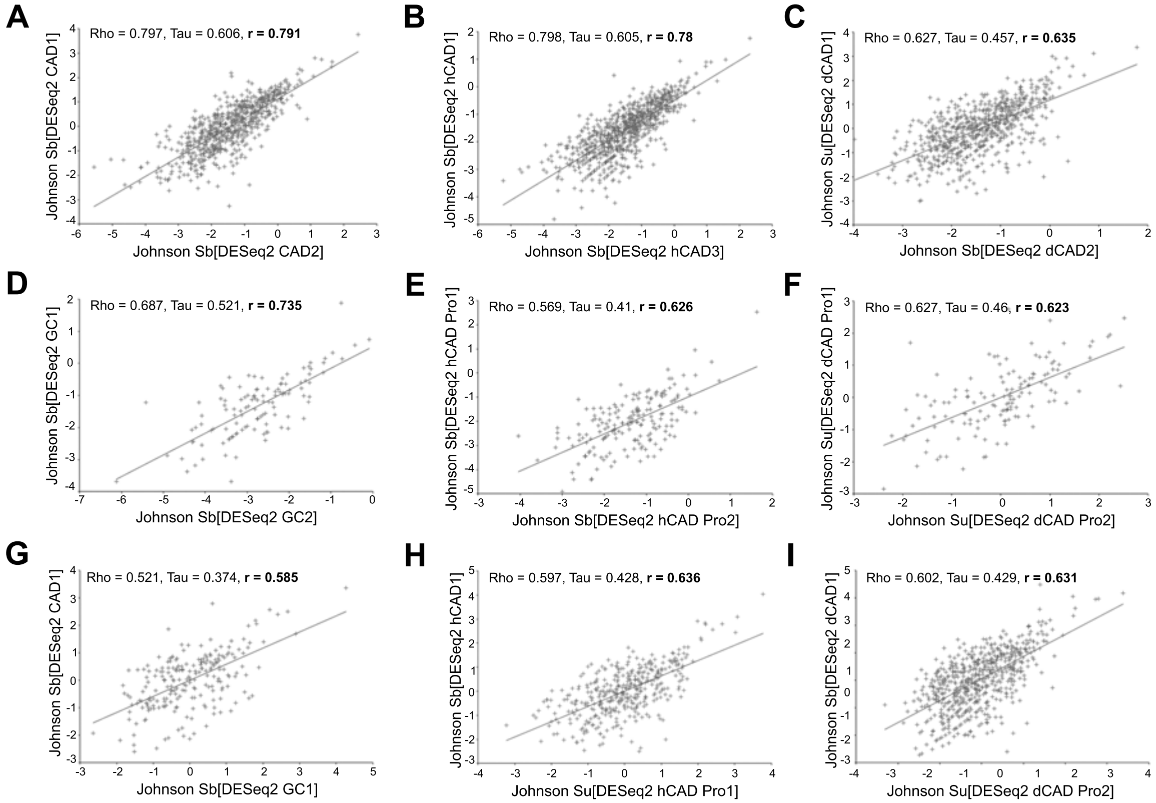

J

VAR vs. VAR	N	Rho	p-value	Pearson R	p-value
CAD 1 VS CAD 2	760	0.797	0	0.791	0
CAD 2 VS CAD 3	690	0.782	0	0.786	0
CAD 1 VS CAD 3	792	0.789	0	0.786	0
**AVERAGE/EDGINGTON'S METHOD**		**0.790**	**0**	**0.788**	**0**
hCAD 1 VS hCAD 2	1131	0.771	0	0.759	0
hCAD 2 VS hCAD 3	1234	0.826	0	0.808	0
hCAD 1 VS hCAD 3	889	0.798	0	0.780	0
**AVERAGE/EDGINGTON'S METHOD**		**0.798**	**0**	**0.783**	**0**
dCAD 1 VS dCAD 2	727	**0.627**	**0**	**0.635**	**0**
GC 1 vs GC 2	116	0.687	1.65E-17	0.735	0
GC 2 vs GC 3	99	0.533	1.37E-08	0.612	9.39E-11
GC 1 vs GC 3	158	0.596	1.40E-16	0.616	6.72E-18
**AVERAGE/EDGINGTON'S METHOD**		**0.605**	**8.61E-25**	**0.655**	**2.76E-31**
hCAD Prot 1 vs hCAD Prot 2	189	**0.569**	**1.37E-17**	**0.626**	**0**
dCAD Prot 1 vs dCADProt 2	138	**0.627**	**1.87E-16**	**0.623**	**3.60E-16**
Neg Con 1 vs Neg Con 2	10	**0.564**	0.0897	**0.629**	5.14E-02
CAD 1 vs. NEGCON 1	15	0.511	0.0517	0.543	3.63E-02
CAD 1 vs. NEGCON 2	46	0.168	0.2650	0.257	8.41E-02
CAD 2 vs. NEGCON 1	19	0.535	0.0182	0.588	8.14E-03
CAD 2 vs. NEGCON 2	55	0.203	0.1365	0.287	3.35E-02
CAD 3 vs. NEGCON 1	22	0.497	0.0185	0.589	3.89E-03
CAD 3 vs. NEGCON 2	50	0.175	0.2233	0.292	3.94E-02
**AVERAGE/EDGINGTON'S METHOD**		**0.348**	**0.0219**	**0.426**	**1.25E-05**

VAR vs. VAR	N	Rho	p-value	Pearson R	p-value
CAD 1 vs. GC 1	224	0.521	5.40E-17	0.585	0
CAD 1 vs. GC 2	123	0.300	7.42E-04	0.404	3.64E-06
CAD 1 vs. GC 3	228	0.509	1.89E-16	0.524	1.68E-17
CAD 2 vs. GC 1	225	0.592	0	0.645	0
CAD 2 vs. GC 2	128	0.346	6.23E-05	0.459	5.08E-08
CAD 2 vs. GC 3	242	0.491	4.27E-16	0.555	0
CAD 3 vs. GC 1	234	0.612	0	0.657	0
CAD 3 vs. GC 2	128	0.434	2.99E-07	0.536	6.92E-11
CAD 3 vs. GC 3	234	0.502	2.52E-16	0.564	0
**AVERAGE/EDGINGTON'S METHOD**		**0.479**	**1.57E-29**	**0.548**	**1.40E-50**
hCAD 1 vs. hCAD Protrusions 1	410	0.597	0	0.636	0
hCAD 1 vs. hCAD Protrusions 2	257	0.539	0	0.557	0
hCAD 2 vs. hCAD Protrusions 1	464	0.627	0	0.634	0
hCAD 2 vs. hCAD Protrusions 2	280	0.453	1.37E-15	0.430	4.71E-14
hCAD 3 vs. hCAD Protrusions 1	431	0.658	0	0.692	0
hCAD 3 vs. hCAD Protrusions 2	264	0.495	1.01E-17	0.490	2.22E-17
**AVERAGE/EDGINGTON'S METHOD**		**0.561**	**1.14E-90**	**0.573**	**1.82E-81**
dCAD 1 vs. dCAD Protrusions 1	159	0.467	5.57E-10	0.464	7.12E-10
dCAD 1 vs. dCAD Protrusions 2	657	0.602	0	0.631	0
dCAD 2 vs. dCAD Protrusions 1	123	0.604	1.42E-13	0.614	4.11E-14
dCAD 2 vs. dCAD Protrusions 2	406	0.659	0	0.668	0
**AVERAGE/EDGINGTON'S METHOD**		**0.583**	**2.41E-38**	**0.594**	**0**
CAD 1 vs. TNT	12	0.322	0.3079	0.392	0.2080
CAD 2 vs. TNT	10	0.697	0.0251	0.743	0.0138
CAD 3 vs. TNT	9	0.817	0.0072	0.810	0.0081
**AVERAGE/EDGINGTON'S METHOD**		**0.612**	**0.0131**	**0.648**	**0.0041**

Figure S2: Pearson Correlations of LCM/MS data. Normalized data (NSAF/DESeq/Johnson transformation) for each MS sample were determined and graphical representations of Pearson correlation between representative replicate samples are shown for (A) CADs, (B) hCADs, (C) dCADs (D) GCs, (E) hCADs protrusions, (F) dCAD protrusions (G) CADs vs GCs, (H) hCADs vs hCAD protrusions and (I) dCADs vs dCAD protrusions. (J) A summary of all Pearson correlation values is shown. Overall, we obtained strong (r>0.6) and moderately strong correlations (0.55<r<0.6) with the LCM/MS samples. As expected, weaker correlations were observed with the negative controls when compared to CADs (r<0.43). P-values <0.05 were considered statistically significant.
