# Supplementary material for: A novel Microproteomic Approach Using Laser Capture Microdissection to Study Cellular Protrusions
Source: Int J Mol Sci. 2019 Mar 7;20(5):1172. doi: 10.3390/ijms20051172 (PMC6429397; doi:10.3390/ijms20051172)
Supplement: Supplementary file 1 [file ijms-20-01172-s001.zip › New-Fig S5G-s.pdf]

G

UNIQUE TNT TERMS	EXCLUSIVE TNT TERMS
RNA N6-methyladenosine methyltransferase complex (GO:0036396)	sublamina densa (GO:0061618)
sublamina densa (GO:0061618)	dendritic branch (GO:0044307)
SMN-Gemin2 complex (GO:0034718)	PAN complex (GO:0031251)
mRNA editing complex (GO:0045293)	interphotoreceptor matrix (GO:0033165)
dendritic branch (GO:0044307)	activin receptor complex (GO:0048179)
PAN complex (GO:0031251)	postsynaptic recycling endosome (GO:0098837)
interphotoreceptor matrix (GO:0033165)	axonal spine (GO:0044308)
activin receptor complex (GO:0048179)	stereocilia ankle link complex (GO:0002142)
delta DNA polymerase complex (GO:0043625)	MutLalpha complex (GO:0032389)
postsynaptic recycling endosome (GO:0098837)	USH2 complex (GO:1990696)
axonal spine (GO:0044308)	intrinsic component of postsynaptic density membrane (GO:0099146)
stereocilia ankle link complex (GO:0002142)	integral component of postsynaptic density membrane (GO:0099061)
MutLalpha complex (GO:0032389)	stereocilia ankle link (GO:0002141)
USH2 complex (GO:1990696)	dense body (GO:0097433)
intrinsic component of postsynaptic density membrane (GO:0099146)	periciliary membrane compartment (GO:1990075)
integral component of postsynaptic density membrane (GO:0099061)	intrinsic component of postsynaptic specialization membrane (GO:0098948)
stereocilia ankle link (GO:0002141)	integral component of postsynaptic specialization membrane (GO:0099060)
dense body (GO:0097433)	stereocilia coupling link (GO:0002139)
periciliary membrane compartment (GO:1990075)	integral component of lumenal side of endoplasmic reticulum membrane (GO:0071556)
intrinsic component of postsynaptic specialization membrane (GO:0098948)	integral component of cytoplasmic side of endoplasmic reticulum membrane (GO:0071458)
integral component of postsynaptic specialization membrane (GO:0099060)	lumenal side of endoplasmic reticulum membrane (GO:0098553)
stereocilia coupling link (GO:0002139)	pericentriolar material (GO:0000242)
integral component of lumenal side of endoplasmic reticulum membrane (GO:0071556)	pi-body (GO:0071546)
integral component of cytoplasmic side of endoplasmic reticulum membrane (GO:0071458)	postsynaptic endosome (GO:0098845)
lumenal side of endoplasmic reticulum membrane (GO:0098553)	dendritic spine membrane (GO:0032591)
pericentriolar material (GO:0000242)	endoplasmic reticulum-Golgi intermediate compartment membrane (GO:0033116)
phagocytic vesicle membrane (GO:0030670)	mismatch repair complex (GO:0032300)
pi-body (GO:0071546)	neurofilament (GO:0005883)
postsynaptic endosome (GO:0098845)	inner dynein arm (GO:0036156)
dendritic spine membrane (GO:0032591)	cytoplasmic side of endoplasmic reticulum membrane (GO:0098554)
endoplasmic reticulum-Golgi intermediate compartment membrane (GO:0033116)	ciliary membrane (GO:0060170)
mismatch repair complex (GO:0032300)	recycling endosome (GO:0055037)
DNA repair complex (GO:1990391)	condensed chromosome, centromeric region (GO:0000779)
cis-Golgi network (GO:0005801)	receptor complex (GO:0043235)
ciliary membrane (GO:0060170)	endoplasmic reticulum-Golgi intermediate compartment (GO:0005793)
condensed chromosome, centromeric region (GO:0000779)	ciliary transition zone (GO:0035869)
recycling endosome (GO:0055037)	neuromuscular junction (GO:0031594)
receptor complex (GO:0043235)	integral component of endoplasmic reticulum membrane (GO:0030176)
neuron projection membrane (GO:0032589)	intrinsic component of endoplasmic reticulum membrane (GO:0031227)
photoreceptor cell cilium (GO:0097733)	ciliary part (GO:0044441)
ciliary part (GO:0044441)	plasma membrane bounded cell projection cytoplasm (GO:0032838)
9+0 non-motile cilium (GO:0097731)	endoplasmic reticulum part (GO:0044432)
plasma membrane bounded cell projection cytoplasm (GO:0032838)	cell projection membrane (GO:0031253)
axoneme (GO:0005930)	endoplasmic reticulum membrane (GO:0005789)
ciliary plasm (GO:0097014)	cilium (GO:0005929)
integral component of endoplasmic reticulum membrane (GO:0030176)	nuclear outer membrane-endoplasmic reticulum membrane network (GO:0042175)
non-motile cilium (GO:0097730)	endoplasmic reticulum subcompartment (GO:0098827)
intrinsic component of endoplasmic reticulum membrane (GO:0031227)	endoplasmic reticulum (GO:0005783)
Golgi membrane (GO:0000139)	plasma membrane (GO:0005886)
endoplasmic reticulum-Golgi intermediate compartment (GO:0005793)	cell periphery (GO:0071944)

Figure S5
