# Supplementary material for: A novel Microproteomic Approach Using Laser Capture Microdissection to Study Cellular Protrusions
Source: Int J Mol Sci. 2019 Mar 7;20(5):1172. doi: 10.3390/ijms20051172 (PMC6429397; doi:10.3390/ijms20051172)
Supplement: Supplementary file 1 [file ijms-20-01172-s001.zip › Sup-Fig-page1-s.pdf]

A novel microproteomic approach using laser capture microdissection to study cellular protrusions

Karine Gousset *, Ana Gordon, Shravan Kumar Kannan and Joey Tovar

Supplementary Materials: includes 6 supplementary figures and legends
