# Supplementary material for: A novel Microproteomic Approach Using Laser Capture Microdissection to Study Cellular Protrusions
Source: Int J Mol Sci. 2019 Mar 7;20(5):1172. doi: 10.3390/ijms20051172 (PMC6429397; doi:10.3390/ijms20051172)
Supplement: Supplementary file 1 [file ijms-20-01172-s001.zip › New-Fig S5C-s.pdf]

C

UNIQUE dCAD TERMS	dCAD IN 2 TERMS	EXCLUSIVE dCAD TERMS
intracellular organelle part (GO:0044446)	**extracellular exosome (GO:0070062)**	organellar large ribosomal subunit (GO:0000315)
organelle part (GO:0044422)	**extracellular vesicle (GO:1903561)**	mitochondrial large ribosomal subunit (GO:0005762)
cytoplasm (GO:0005737)	**extracellular organelle (GO:0043230)**	mitochondrion (GO:0005739)
organelle (GO:0043226)	ribonucleoprotein complex (GO:1990904)	cytoplasm (GO:0005737)
intracellular part (GO:0044424)	intracellular ribonucleoprotein complex (GO:0030529)	organellar ribosome (GO:0000313)
intracellular (GO:0005622)	**myelin sheath (GO:0043209)**	mitochondrial ribosome (GO:0005761)
cytoplasmic part (GO:0044444)	macromolecular complex (GO:0032991)	intracellular part (GO:0044424)
**intracellular membrane-bounded organelle (GO:0043231)**	proteasome regulatory particle (GO:0005838)	cytoplasmic part (GO:0044444)
macromolecular complex (GO:0032991)	proteasome complex (GO:0000502)	intracellular (GO:0005622)
cell part (GO:0044464)	endopeptidase complex (GO:1905369)	organelle (GO:0043226)
cell (GO:0005623)	eukaryotic translation initiation factor 3 complex (GO:0005852)	intracellular organelle (GO:0043229)
ribonucleoprotein complex (GO:1990904)	cytosolic large ribosomal subunit (GO:0022625)	**membrane-bounded organelle (GO:0043227)**
intracellular ribonucleoprotein complex (GO:0030529)	cytosol (GO:0005829)	mitochondrial part (GO:0044429)
intracellular organelle lumen (GO:0070013)	proteasome accessory complex (GO:0022624)	**intracellular membrane-bounded organelle (GO:0043231)**
**membrane-enclosed lumen (GO:0031974)**	cytosolic ribosome (GO:0022626)	uniplex complex (GO:1990246)
organelle lumen (GO:0043233)	**extracellular region part (GO:0044421)**	mitochondrial inner membrane protein insertion complex (GO:0042721)
cytosol (GO:0005829)	**extracellular space (GO:0005615)**	mitochondrial matrix (GO:0005759)
protein complex (GO:0043234)	**vesicle (GO:0031982)**	cell part (GO:0044464)
**extracellular exosome (GO:0070062)**	cytosolic part (GO:0044445)	cell (GO:0005623)
**extracellular vesicle (GO:1903561)**	aminoacyl-tRNA synthetase multienzyme complex (GO:0017101)	Hrd1p ubiquitin ligase ERAD-L complex (GO:0000839)
**extracellular organelle (GO:0043230)**	intracellular organelle part (GO:0044446)	ciliary pocket membrane (GO:0020018)
nuclear part (GO:0044428)	organelle part (GO:0044422)	ciliary pocket (GO:0020016)
mitochondrion (GO:0005739)	cytoplasmic part (GO:0044444)	m-AAA complex (GO:0005745)
nucleus (GO:0005634)	proteasome regulatory particle, base subcomplex (GO:0008540)	intracellular organelle part (GO:0044446)
intracellular non-membrane-bounded organelle (GO:0043232)	protein complex (GO:0043234)	organelle part (GO:0044422)
non-membrane-bounded organelle (GO:0043228)	intracellular part (GO:0044424)	mitochondrial inner membrane (GO:0005743)
nuclear lumen (GO:0031981)	intracellular (GO:0005622)	mitochondrial membrane (GO:0031966)
**vesicle (GO:0031982)**	peptidase complex (GO:1905368)	phosphatidylinositol 3-kinase complex, class III, type II (GO:0034272)
mitochondrial part (GO:0044429)	cytoplasm (GO:0005737)	phosphatidylinositol 3-kinase complex, class III, type I (GO:0034271)
organelle envelope (GO:0031967)	ribosome (GO:0005840)	calcineurin complex (GO:0005955)
envelope (GO:0031975)	**extracellular region (GO:0005576)**	organelle membrane (GO:0031090)
nucleoplasm (GO:0005654)	**membrane-bounded organelle (GO:0043227)**	mitochondrial envelope (GO:0005740)
catalytic complex (GO:1902494)	**focal adhesion (GO:0005925)**	organelle inner membrane (GO:0019866)
organelle membrane (GO:0031090)	organelle (GO:0043226)	**clathrin complex (GO:0071439)**
nucleolus (GO:0005730)	**cell-substrate adherens junction (GO:0005924)**	sorting endosome (GO:0097443)
spliceosomal complex (GO:0005681)	**cell-substrate junction (GO:0030055)**	GPI-anchor transamidase complex (GO:0042765)
mitochondrial membrane (GO:0031966)	nuclear proteasome complex (GO:0031595)	Hrd1p ubiquitin ligase complex (GO:0000836)
mitochondrial envelope (GO:0005740)	eukaryotic translation initiation factor 3 complex, eIF3m (GO:0071541)	ER ubiquitin ligase complex (GO:0000835)
mitochondrial inner membrane (GO:0005743)	intracellular organelle (GO:0043229)	protein phosphatase 4 complex (GO:0030289)
organelle inner membrane (GO:0019866)	chaperonin-containing T-complex (GO:0005832)	organelle envelope (GO:0031967)
**membrane protein complex (GO:0098796)**	cytosolic proteasome complex (GO:0031597)	envelope (GO:0031975)
catalytic step 2 spliceosome (GO:0071013)	**intracellular membrane-bounded organelle (GO:0043231)**	ESCRT complex (GO:0036452)
mitochondrial matrix (GO:0005759)	chaperone complex (GO:0101031)	meiotic spindle (GO:0072687)
endopeptidase complex (GO:1905369)	**adherens junction (GO:0005912)**	astral microtubule (GO:0000235)
proteasome complex (GO:0000502)	intracellular organelle lumen (GO:0070013)	signal peptidase complex (GO:0005787)
mitochondrial protein complex (GO:0098798)	**membrane-enclosed lumen (GO:0031974)**	Bcl-2 family protein complex (GO:0097136)
**extracellular space (GO:0005615)**	organelle lumen (GO:0043233)	Mre11 complex (GO:0030870)
peptidase complex (GO:1905368)	**anchoring junction (GO:0070161)**	mitochondrial membrane part (GO:0044455)
**extracellular region part (GO:0044421)**	ribosomal subunit (GO:0044391)	integral component of mitochondrial membrane (GO:0032592)
endomembrane system (GO:0012505)	**zona pellucida receptor complex (GO:0002199)**	**membrane-enclosed lumen (GO:0031974)**

Figure S5
