# Supplementary material for: A novel Microproteomic Approach Using Laser Capture Microdissection to Study Cellular Protrusions
Source: Int J Mol Sci. 2019 Mar 7;20(5):1172. doi: 10.3390/ijms20051172 (PMC6429397; doi:10.3390/ijms20051172)
Supplement: Supplementary file 1 [file ijms-20-01172-s001.zip › New-Fig S5A-s.pdf]

A

UNIQUE CAD TERMS	CAD IN 2 TERMS	EXCLUSIVE CAD TERMS
ribonucleoprotein complex (GO:1990904)	ribonucleoprotein complex (GO:1990904)	actomyosin contractile ring (GO:0005826)
intracellular ribonucleoprotein complex (GO:0030529)	intracellular ribonucleoprotein complex (GO:0030529)	chromatin silencing complex (GO:0005677)
macromolecular complex (GO:0032991)	**myelin sheath (GO:0043209)**	bleb (GO:0032059)
intracellular organelle part (GO:0044446)	**extracellular exosome (GO:0070062)**	MCM8-MCM9 complex (GO:0097362)
**extracellular exosome (GO:0070062)**	**extracellular vesicle (GO:1903561)**	apoptosome (GO:0043293)
**extracellular vesicle (GO:1903561)**	**extracellular organelle (GO:0043230)**	haptoglobin-hemoglobin complex (GO:0031838)
**extracellular organelle (GO:0043230)**	macromolecular complex (GO:0032991)	**myosin II complex (GO:0016460)**
organelle part (GO:0044422)	intracellular organelle part (GO:0044446)	translocon complex (GO:0071256)
intracellular part (GO:0044424)	organelle part (GO:0044422)	Sec61 translocon complex (GO:0005784)
intracellular (GO:0005622)	proteasome complex (GO:0000502)	contractile ring (GO:0070938)
organelle (GO:0043226)	endopeptidase complex (GO:1905369)	muscle myosin complex (GO:0005859)
intracellular organelle (GO:0043229)	**vesicle (GO:0031982)**	smooth muscle contractile fiber (GO:0030485)
**membrane-bounded organelle (GO:0043227)**	cytosol (GO:0005829)	myosin filament (GO:0032982)
cytoplasm (GO:0005737)	intracellular part (GO:0044424)	**kinesin complex (GO:0005871)**
**intracellular membrane-bounded organelle (GO:0043231)**	intracellular (GO:0005622)	**neuronal ribonucleoprotein granule (GO:0071598)**
cytoplasmic part (GO:0044444)	cytosolic part (GO:0044445)	CCR4-NOT core complex (GO:0030015)
cytosol (GO:0005829)	cytoplasm (GO:0005737)	kinetochore microtubule (GO:0005828)
protein complex (GO:0043234)	**extracellular space (GO:0005615)**	myosin complex (GO:0016459)
intracellular organelle lumen (GO:0070013)	**extracellular region part (GO:0044421)**	rough endoplasmic reticulum membrane (GO:0030867)
**membrane-enclosed lumen (GO:0031974)**	cytosolic large ribosomal subunit (GO:0022625)	**microtubule associated complex (GO:0005875)**
organelle lumen (GO:0043233)	protein complex (GO:0043234)	**signal recognition particle receptor complex (GO:0005785)**
nuclear part (GO:0044428)	**membrane-bounded organelle (GO:0043227)**	HAUS complex (GO:0070652)
intracellular non-membrane-bounded organelle (GO:0043232)	cytosolic ribosome (GO:0022626)	nuclear transcriptional repressor complex (GO:0090568)
non-membrane-bounded organelle (GO:0043228)	spliceosomal complex (GO:0005681)	**outer dynein arm (GO:0036157)**
**vesicle (GO:0031982)**	cytoplasmic part (GO:0044444)	mitochondrial proton-transporting ATP synthase complex, coupling factor F(o) (GO:0000276)
nucleus (GO:0005634)	intracellular organelle (GO:0043229)	MCM complex (GO:0042555)
cell part (GO:0044464)	proteasome accessory complex (GO:0022624)	nuclear exosome (RNase complex) (GO:0000176)
cell (GO:0005623)	organelle (GO:0043226)	condensed nuclear chromosome kinetochore (GO:0000778)
**myelin sheath (GO:0043209)**	proteasome regulatory particle (GO:0005838)	**cortical actin cytoskeleton (GO:0030864)**
nuclear lumen (GO:0031981)	eukaryotic translation initiation factor 3 complex (GO:0005852)	cell division site part (GO:0032155)
spliceosomal complex (GO:0005681)	**intracellular membrane-bounded organelle (GO:0043231)**	cell division site (GO:0032153)
**extracellular space (GO:0005615)**	peptidase complex (GO:1905368)	**contractile actin filament bundle (GO:0097517)**
**extracellular region part (GO:0044421)**	**extracellular region (GO:0005576)**	stress fiber (GO:0001725)
proteasome complex (GO:0000502)	aminoacyl-tRNA synthetase multienzyme complex (GO:0017101)	**actomyosin (GO:0042641)**
endopeptidase complex (GO:1905369)	nucleus (GO:0005634)	**cornified envelope (GO:0001533)**
catalytic step 2 spliceosome (GO:0071013)	intracellular organelle lumen (GO:0070013)	rough endoplasmic reticulum (GO:0005791)
**extracellular region (GO:0005576)**	**membrane-enclosed lumen (GO:0031974)**	contractile fiber (GO:0043292)
nucleolus (GO:0005730)	organelle lumen (GO:0043233)	**cytoplasmic side of plasma membrane (GO:0009898)**
peptidase complex (GO:1905368)	nuclear part (GO:0044428)	**cytoplasmic side of membrane (GO:0098562)**
mitochondrion (GO:0005739)	catalytic step 2 spliceosome (GO:0071013)	supramolecular fiber (GO:0099512)
proteasome accessory complex (GO:0022624)	cell part (GO:0044464)	supramolecular polymer (GO:0099081)
proteasome regulatory particle (GO:0005838)	cell (GO:0005623)	supramolecular complex (GO:0099080)
eukaryotic translation initiation factor 3 complex (GO:0005852)	eukaryotic translation initiation factor 3 complex, eIF3m (GO:0071541)	**actin filament bundle (GO:0032432)**
spliceosomal snRNP complex (GO:0097525)	**focal adhesion (GO:0005925)**	transcriptional repressor complex (GO:0017053)
**focal adhesion (GO:0005925)**	ribosome (GO:0005840)	myofibril (GO:0030016)
nucleoplasm (GO:0005654)	**cell-substrate adherens junction (GO:0005924)**	**cortical cytoskeleton (GO:0030863)**
aminoacyl-tRNA synthetase multienzyme complex (GO:0017101)	**cell-substrate junction (GO:0030055)**	**lateral plasma membrane (GO:0016328)**
**cell-substrate adherens junction (GO:0005924)**	proteasome core complex (GO:0005839)	**microtubule (GO:0005874)**
**cell-substrate junction (GO:0030055)**	nuclear lumen (GO:0031981)	cytoplasmic region (GO:0099568)
small nuclear ribonucleoprotein complex (GO:0030532)	intracellular non-membrane-bounded organelle (GO:0043232)	contractile fiber part (GO:0044449)

Figure S5
