# Supplementary material for: A novel Microproteomic Approach Using Laser Capture Microdissection to Study Cellular Protrusions
Source: Int J Mol Sci. 2019 Mar 7;20(5):1172. doi: 10.3390/ijms20051172 (PMC6429397; doi:10.3390/ijms20051172)
Supplement: Supplementary file 1 [file ijms-20-01172-s001.zip › New-Fig S5F-s.pdf]

F

UNIQUE GC TERMS	GC IN 2 TERMS	EXCLUSIVE GC TERMS
cytosolic ribosome (GO:0022626)	cytosolic ribosome (GO:0022626)	perineuronal net (GO:0072534)
myelin sheath (GO:0043209)	chaperonin-containing T-complex (GO:0005832)	muscle thin filament tropomyosin (GO:0005862)
extracellular exosome (GO:0070062)	chaperone complex (GO:0101031)	terminal web (GO:1990357)
extracellular vesicle (GO:1903561)	zona pellucida receptor complex (GO:0002199)	microtubule bundle (GO:0097427)
extracellular organelle (GO:0043230)	cytosolic small ribosomal subunit (GO:0022627)	striated muscle thin filament (GO:0005865)
cytosolic part (GO:0044445)	cytosolic part (GO:0044445)	BORC complex (GO:0099078)
cytosolic small ribosomal subunit (GO:0022627)	ribosomal subunit (GO:0044391)	axonemal microtubule (GO:0005879)
ribosomal subunit (GO:0044391)	ribosome (GO:0005840)	phosphopyruvate hydratase complex (GO:0000015)
ribosome (GO:0005840)	small ribosomal subunit (GO:0015935)	myofilament (GO:0036379)
cytosolic large ribosomal subunit (GO:0022625)	myelin sheath (GO:0043209)	dendritic growth cone (GO:0044294)
small ribosomal subunit (GO:0015935)	cytosolic large ribosomal subunit (GO:0022625)	axoneme part (GO:0044447)
extracellular space (GO:0005615)	extracellular exosome (GO:0070062)	outer dynein arm (GO:0036157)
chaperonin-containing T-complex (GO:0005832)	extracellular vesicle (GO:1903561)	HOPS complex (GO:0030897)
vesicle (GO:0031982)	extracellular organelle (GO:0043230)	AP-3 adaptor complex (GO:0030123)
extracellular region part (GO:0044421)	focal adhesion (GO:0005925)	phagophore assembly site membrane (GO:0034045)
chaperone complex (GO:0101031)	cell-substrate adherens junction (GO:0005924)	voltage-gated sodium channel complex (GO:0001518)
focal adhesion (GO:0005925)	cell-substrate junction (GO:0030055)	ciliary rootlet (GO:0035253)
cell-substrate adherens junction (GO:0005924)	ribonucleoprotein complex (GO:1990904)	axon initial segment (GO:0043194)
cell-substrate junction (GO:0030055)	intracellular ribonucleoprotein complex (GO:0030529)	BLOC-1 complex (GO:0031083)
ribonucleoprotein complex (GO:1990904)	endoplasmic reticulum chaperone complex (GO:0034663)	node of Ranvier (GO:0033268)
intracellular ribonucleoprotein complex (GO:0030529)	adherens junction (GO:0005912)	dendrite terminus (GO:0044292)
zona pellucida receptor complex (GO:0002199)	anchoring junction (GO:0070161)	paranode region of axon (GO:0033270)
extracellular region (GO:0005576)	extracellular matrix (GO:0031012)	actin filament (GO:0005884)
cytosol (GO:0005829)	extracellular region part (GO:0044421)	axonemal dynein complex (GO:0005858)
adherens junction (GO:0005912)	extracellular space (GO:0005615)	BLOC complex (GO:0031082)
anchoring junction (GO:0070161)	large ribosomal subunit (GO:0015934)	sodium channel complex (GO:0034706)
proteasome core complex, alpha-subunit complex (GO:0019773)	vesicle (GO:0031982)	phagophore assembly site (GO:0000407)
large ribosomal subunit (GO:0015934)	extracellular region (GO:0005576)	axoneme (GO:0005930)
proteasome core complex (GO:0005839)	presynaptic cytosol (GO:0099523)	ciliary plasm (GO:0097014)
extracellular matrix (GO:0031012)	citrate lyase complex (GO:0009346)	axon part (GO:0033267)
macromolecular complex (GO:0032991)	inactive sex chromosome (GO:0098577)	polymeric cytoskeletal fiber (GO:0099513)
UBC13-UEV1A complex (GO:0035370)	cytosol (GO:0005829)	plasma membrane bounded cell projection cytoplasm (GO:0032838
mitotic spindle microtubule (GO:1990498)	type III intermediate filament (GO:0045098)	sarcolemma (GO:0042383)
methionine adenosyltransferase complex (GO:0048269)	smooth endoplasmic reticulum (GO:0005790)	lytic vacuole (GO:0000323)
polysome (GO:0005844)	VCP-NSFL1C complex (GO:1990730)	lysosome (GO:0005764)
cytoplasmic part (GO:0044444)	lysosomal matrix (GO:1990836)	cluster of actin-based cell projections (GO:0098862)
cytoplasm (GO:0005737)	mitotic spindle microtubule (GO:1990498)	axon (GO:0030424)
eukaryotic translation initiation factor 2 complex (GO:0005850)	NF-kappaB complex (GO:0071159)	sarcomere (GO:0030017)
endoplasmic reticulum chaperone complex (GO:0034663)	sperm mitochondrial sheath (GO:0097226)	cytoplasmic side of plasma membrane (GO:0009898)
intracellular non-membrane-bounded organelle (GO:0043232)	RNA nuclear export complex (GO:0042565)	growth cone (GO:0030426)
non-membrane-bounded organelle (GO:0043228)	IRE1-RACK1-PP2A complex (GO:1990630)	site of polarized growth (GO:0030427)
microspike (GO:0044393)	cytoplasmic part (GO:0044444)	vacuole (GO:0005773)
Schwann cell microvillus (GO:0097454)	prefoldin complex (GO:0016272)	cytoplasmic side of membrane (GO:0098562)
Myb complex (GO:0031523)	intracellular non-membrane-bounded organelle (GO:0043232)	actin-based cell projection (GO:0098858)
eukaryotic translation elongation factor 1 complex (GO:0005853)	non-membrane-bounded organelle (GO:0043228)	supramolecular fiber (GO:0099512)
nucleocytoplasmic transport complex (GO:0031074)	macromolecular complex (GO:0032991)	supramolecular polymer (GO:0099081)
intracellular organelle part (GO:0044446)	messenger ribonucleoprotein complex (GO:1990124)	supramolecular complex (GO:0099080)
organelle part (GO:0044422)	actin filament bundle (GO:0032432)	contractile fiber part (GO:0044449)
organelle (GO:0043226)	proteasome core complex, alpha-subunit complex (GO:0019773)	myofibril (GO:0030016)
intracellular part (GO:0044424)	cytoplasm (GO:0005737)	cell projection part (GO:0044463)

Figure S5
