# Supplementary material for: A novel Microproteomic Approach Using Laser Capture Microdissection to Study Cellular Protrusions
Source: Int J Mol Sci. 2019 Mar 7;20(5):1172. doi: 10.3390/ijms20051172 (PMC6429397; doi:10.3390/ijms20051172)
Supplement: Supplementary file 1 [file ijms-20-01172-s001.zip › New-Fig S5D-s.pdf]

D

UNIQUE hCAD PROTRUSION TERMS	hCAD PROTRUSION IN 2 TERMS	EXCLUSIVE hCAD PROTRUSION TERMS
**myelin sheath (GO:0043209)**	cytosolic ribosome (GO:0022626)	calcium- and calmodulin-dependent protein kinase complex (GO:0005954)
**extracellular exosome (GO:0070062)**	**myelin sheath (GO:0043209)**	RAVE complex (GO:0043291)
**extracellular vesicle (GO:1903561)**	cytosolic small ribosomal subunit (GO:0022627)	FHF complex (GO:0070695)
**extracellular organelle (GO:0043230)**	cytosolic part (GO:0044445)	**dendritic spine neck (GO:0044326)**
ribonucleoprotein complex (GO:1990904)	chaperonin-containing T-complex (GO:0005832)	sodium:potassium-exchanging ATPase complex (GO:0005890)
intracellular ribonucleoprotein complex (GO:0030529)	ribosomal subunit (GO:0044391)	**neurofilament (GO:0005883)**
cytosolic part (GO:0044445)	chaperone complex (GO:0101031)	**cell tip (GO:0051286)**
cytosolic ribosome (GO:0022626)	ribosome (GO:0005840)	cation-transporting ATPase complex (GO:0090533)
proteasome complex (GO:0000502)	**zona pellucida receptor complex (GO:0002199)**	**dendritic spine head (GO:0044327)**
eukaryotic translation initiation factor 3 complex (GO:0005852)	small ribosomal subunit (GO:0015935)	ATPase dependent transmembrane transport complex (GO:0098533)
endopeptidase complex (GO:1905369)	cytosolic large ribosomal subunit (GO:0022625)	sperm fibrous sheath (GO:0035686)
**vesicle (GO:0031982)**	**extracellular exosome (GO:0070062)**	eukaryotic translation initiation factor 3 complex (GO:0005852)
**focal adhesion (GO:0005925)**	**extracellular vesicle (GO:1903561)**	**dendritic spine (GO:0043197)**
**extracellular space (GO:0005615)**	**extracellular organelle (GO:0043230)**	**neuron spine (GO:0044309)**
**extracellular region part (GO:0044421)**	**focal adhesion (GO:0005925)**	sperm principal piece (GO:0097228)
**cell-substrate adherens junction (GO:0005924)**	**cell-substrate adherens junction (GO:0005924)**	mitotic spindle pole (GO:0097431)
macromolecular complex (GO:0032991)	**cell-substrate junction (GO:0030055)**	**postsynapse (GO:0098794)**
**cell-substrate junction (GO:0030055)**	**adherens junction (GO:0005912)**	**growth cone (GO:0030426)**
proteasome accessory complex (GO:0022624)	**anchoring junction (GO:0070161)**	**site of polarized growth (GO:0030427)**
proteasome regulatory particle (GO:0005838)	**VCP-NSFL1C complex (GO:1990730)**	**synapse part (GO:0044456)**
proteasome core complex (GO:0005839)	mitotic spindle microtubule (GO:1990498)	**ciliary part (GO:0044441)**
nuclear proteasome complex (GO:0031595)	**extracellular matrix (GO:0031012)**	**neuronal cell body (GO:0043025)**
cytosol (GO:0005829)	ribonucleoprotein complex (GO:1990904)	**postsynaptic membrane (GO:0045211)**
**extracellular region (GO:0005576)**	intracellular ribonucleoprotein complex (GO:0030529)	**dendrite (GO:0030425)**
**zona pellucida receptor complex (GO:0002199)**	large ribosomal subunit (GO:0015934)	**dendritic tree (GO:0097447)**
proteasome regulatory particle, base subcomplex (GO:0008540)	**extracellular region part (GO:0044421)**	**cell projection part (GO:0044463)**
**adherens junction (GO:0005912)**	**extracellular space (GO:0005615)**	**plasma membrane bounded cell projection part (GO:0120038)**
peptidase complex (GO:1905368)	eukaryotic translation elongation factor 1 complex (GO:0005853)	**somatodendritic compartment (GO:0036477)**
chaperonin-containing T-complex (GO:0005832)	Myb complex (GO:0031523)	**synapse (GO:0045202)**
ribosome (GO:0005840)	**vesicle (GO:0031982)**	cell body (GO:0044297)
**anchoring junction (GO:0070161)**	**extracellular region (GO:0005576)**	**cilium (GO:0005929)**
intracellular organelle part (GO:0044446)	cytosol (GO:0005829)	**neuron projection (GO:0043005)**
chaperone complex (GO:0101031)	proteasome core complex, alpha-subunit complex (GO:0019773)	**plasma membrane bounded cell projection (GO:0120025)**
cytoplasm (GO:0005737)	**type III intermediate filament (GO:0045098)**	**neuron part (GO:0097458)**
organelle part (GO:0044422)	macromolecular complex (GO:0032991)	**integral component of plasma membrane (GO:0005887)**
cytoplasmic part (GO:0044444)	endoplasmic reticulum chaperone complex (GO:0034663)	**intrinsic component of plasma membrane (GO:0031226)**
protein complex (GO:0043234)	polysome (GO:0005844)	**cell projection (GO:0042995)**
cytosolic large ribosomal subunit (GO:0022625)	cytoplasmic part (GO:0044444)	**plasma membrane (GO:0005886)**
ribosomal subunit (GO:0044391)	**cell junction (GO:0030054)**	
proteasome core complex, alpha-subunit complex (GO:0019773)	intracellular non-membrane-bounded organelle (GO:0043232)	
eukaryotic translation initiation factor 3 complex, eIF3m (GO:0071541)	non-membrane-bounded organelle (GO:0043228)	
**membrane-bounded organelle (GO:0043227)**	smooth endoplasmic reticulum (GO:0005790)	
intracellular part (GO:0044424)	glycogen granule (GO:0042587)	
cytosolic proteasome complex (GO:0031597)	eukaryotic translation initiation factor 3 complex (GO:0005852)	
intracellular (GO:0005622)	**tubulin complex (GO:0045298)**	
organelle (GO:0043226)	mitochondrial proton-transporting ATP synthase complex, catalytic core F(1) (GO:0000275)	
intracellular organelle (GO:0043229)	proton-transporting ATP synthase complex, catalytic core F(1) (GO:0045261)	
prefoldin complex (GO:0016272)	cytoplasm (GO:0005737)	
eukaryotic translation elongation factor 1 complex (GO:0005853)	proton-transporting two-sector ATPase complex, catalytic domain (GO:0033178)	
nucleocytoplasmic transport complex (GO:0031074)	protein complex (GO:0043234)	

Figure S5
