# Supplementary material for: A novel Microproteomic Approach Using Laser Capture Microdissection to Study Cellular Protrusions
Source: Int J Mol Sci. 2019 Mar 7;20(5):1172. doi: 10.3390/ijms20051172 (PMC6429397; doi:10.3390/ijms20051172)
Supplement: Supplementary file 1 [file ijms-20-01172-s001.zip › legend-Supfig3-s.pdf]

Figure S3: IF from HPA Subcell database. (A) Examples of IF images obtained from the HPA Subcell database of proteins found within protrusions (white arrows). Image Credit: Human Protein Atlas: https://www.proteinatlas.org/ENSG00000168036-CTNNB1/cell#img; https://www.proteinatlas.org/images/29160/602_B4_1_blue_red_green.jpg; https://www.proteinatlas.org/ENSG00000147065-MSN/cell#img; https://www.proteinatlas.org/images/11135/85_B8_1_blue_red_green.jpg; https://www.proteinatlas.org/ENSG00000092820-EZR/cell#img; https://www.proteinatlas.org/images/21616/193_D2_1_blue_red_green.jpg; https://www.proteinatlas.org/ENSG00000128641-MYO1B/cell; https://www.proteinatlas.org/images/13607/160_F3_2_blue_red_green.jpg; https://www.proteinatlas.org/ENSG00000127603-MACF1/cell#img; http://www.proteinatlas.org/images/68103/1306_D11_2_blue_red_green.jpg; https://www.proteinatlas.org/ENSG00000130396-AFDN/cell#img; https://www.proteinatlas.org/images/30213/327_E9_2_blue_red_green.jpg.
(B) Out of the 8 proteins we analyzed by IF (Fig. 5), 6 were found in HPA. IF of Grk5, Cd47, Hspa1b and Cobl from HPA show that they were found in protrusions (see white arrows) but Tenm2 and Hist1h3b on the HPA images were not found within protrusions. Image Credit: Human Protein Atlas: https://www.proteinatlas.org/ENSG00000198873-GRK5/cell#img; https://www.proteinatlas.org/images/46838/1752_G8_18_cr5804e4de4d29f_red_green.j jp; https://www.proteinatlas.org/ENSG00000196776-CD47/cell#img; https://www.proteinatlas.org/images/16055/204_F3_2_blue_red_green.jpg; https://www.proteinatlas.org/ENSG00000145934-TENM2/cell#img; https://www.proteinatlas.org/images/68691/1402_D5_1_red_green.jpg; https://www.proteinatlas.org/ENSG00000204388-HSPA1B/cell#img; https://www.proteinatlas.org/images/52504/987_G4_2_blue_red_green.jpg; https://www.proteinatlas.org/ENSG00000106078-COBL/cell#img; https://www.proteinatlas.org/images/19167/1044_C4_2_blue_red_green.jpg; https://www.proteinatlas.org/ENSG00000168148-HIST3H3/cell; https://www.proteinatlas.org/images/3812/624_D5_1_red_green.jpg.
HPA is not optimized for the visualization of protrusions. This suggests that the HPA enrichment analysis (Fig. 5i) likely understates the actual enrichment. If we compare our IF to the HPA, the enrichment may be understated by ~33% (4 out of 6 proteins agreed were found in protrusions using HPA).
