# Supplementary material for: A novel Microproteomic Approach Using Laser Capture Microdissection to Study Cellular Protrusions
Source: Int J Mol Sci. 2019 Mar 7;20(5):1172. doi: 10.3390/ijms20051172 (PMC6429397; doi:10.3390/ijms20051172)
Supplement: Supplementary file 1 [file ijms-20-01172-s001.zip › New-Fig S5B-s.pdf]

B

UNIQUE hCAD TERMS	hCAD IN 2 TERMS	EXCLUSIVE hCAD TERMS
intracellular organelle part (GO:0044446)	ribonucleoprotein complex (GO:1990904)	RZZ complex (GO:1990423)
organelle part (GO:0044422)	intracellular ribonucleoprotein complex (GO:0030529)	heteromeric SMAD protein complex (GO:0071144)
macromolecular complex (GO:0032991)	macromolecular complex (GO:0032991)	**dendritic spine cytoplasm (GO:0061846)**
intracellular organelle (GO:0043229)	intracellular organelle part (GO:0044446)	**neuron projection branch point (GO:0061845)**
**membrane-bounded organelle (GO:0043227)**	organelle part (GO:0044422)	**presynaptic cytosol (GO:0099523)**
intracellular part (GO:0044424)	**extracellular exosome (GO:0070062)**	striated muscle dense body (GO:0055120)
intracellular (GO:0005622)	**extracellular vesicle (GO:1903561)**	transcription factor TFIIE complex (GO:0005673)
**intracellular membrane-bounded organelle (GO:0043231)**	intracellular part (GO:0044424)	nuclear pore transmembrane ring (GO:0070762)
ribonucleoprotein complex (GO:1990904)	**extracellular organelle (GO:0043230)**	DUBm complex (GO:0071819)
intracellular ribonucleoprotein complex (GO:0030529)	intracellular (GO:0005622)	box H/ACA scaRNP complex (GO:0072589)
cytoplasm (GO:0005737)	**membrane-bounded organelle (GO:0043227)**	transcription export complex 2 (GO:0070390)
nuclear part (GO:0044428)	organelle (GO:0043226)	mature chylomicron (GO:0034359)
**membrane-enclosed lumen (GO:0031974)**	intracellular organelle (GO:0043229)	3-methylcrotonyl-CoA carboxylase complex, mitochondrial (GO:0002169)
organelle lumen (GO:0043233)	cytoplasm (GO:0005737)	THO complex part of transcription export complex (GO:0000445)
intracellular organelle lumen (GO:0070013)	protein complex (GO:0043234)	THO complex (GO:0000347)
cytoplasmic part (GO:0044444)	**intracellular membrane-bounded organelle (GO:0043231)**	transcriptionally active chromatin (GO:0035327)
cell part (GO:0044464)	nuclear part (GO:0044428)	NELF complex (GO:0032021)
cell (GO:0005623)	intracellular organelle lumen (GO:0070013)	Ndc80 complex (GO:0031262)
nucleus (GO:0005634)	**membrane-enclosed lumen (GO:0031974)**	EARP complex (GO:1990745)
protein complex (GO:0043234)	organelle lumen (GO:0043233)	intracellular organelle (GO:0043229)
nuclear lumen (GO:0031981)	cytosol (GO:0005829)	nuclear part (GO:0044428)
intracellular non-membrane-bounded organelle (GO:0043232)	cytoplasmic part (GO:0044444)	transcription elongation factor complex (GO:0008023)
non-membrane-bounded organelle (GO:0043228)	nucleus (GO:0005634)	intracellular organelle part (GO:0044446)
cytosol (GO:0005829)	nuclear lumen (GO:0031981)	intracellular part (GO:0044424)
**extracellular exosome (GO:0070062)**	endopeptidase complex (GO:1905369)	intracellular (GO:0005622)
**extracellular vesicle (GO:1903561)**	intracellular non-membrane-bounded organelle (GO:0043232)	organelle part (GO:0044422)
**extracellular organelle (GO:0043230)**	non-membrane-bounded organelle (GO:0043228)	spliceosomal complex (GO:0005681)
spliceosomal complex (GO:0005681)	cell part (GO:0044464)	alpha DNA polymerase:primase complex (GO:0005658)
nucleoplasm (GO:0005654)	cell (GO:0005623)	SMAD protein complex (GO:0071141)
nucleolus (GO:0005730)	proteasome complex (GO:0000502)	chromosome passenger complex (GO:0032133)
catalytic complex (GO:1902494)	**vesicle (GO:0031982)**	transcription export complex (GO:0000346)
mitochondrion (GO:0005739)	**myelin sheath (GO:0043209)**	**intracellular membrane-bounded organelle (GO:0043231)**
**vesicle (GO:0031982)**	spliceosomal complex (GO:0005681)	organelle (GO:0043226)
organelle envelope (GO:0031967)	peptidase complex (GO:1905368)	nuclear lumen (GO:0031981)
envelope (GO:0031975)	**extracellular space (GO:0005615)**	transcription factor TFIIIC complex (GO:0000127)
preribosome (GO:0030684)	**extracellular region part (GO:0044421)**	Pwp2p-containing subcomplex of 90S preribosome (GO:0034388)
catalytic step 2 spliceosome (GO:0071013)	proteasome accessory complex (GO:0022624)	**membrane-enclosed lumen (GO:0031974)**
mitochondrial part (GO:0044429)	nucleoplasm (GO:0005654)	organelle lumen (GO:0043233)
organelle membrane (GO:0031090)	proteasome regulatory particle (GO:0005838)	nuclear exosome (RNase complex) (GO:0000176)
chromosome (GO:0005694)	nucleolus (GO:0005730)	protein complex (GO:0043234)
endopeptidase complex (GO:1905369)	**focal adhesion (GO:0005925)**	**membrane-bounded organelle (GO:0043227)**
proteasome complex (GO:0000502)	**cell-substrate adherens junction (GO:0005924)**	intracellular organelle lumen (GO:0070013)
nuclear pore (GO:0005643)	**cell-substrate junction (GO:0030055)**	chromosomal region (GO:0098687)
**membrane protein complex (GO:0098796)**	catalytic complex (GO:1902494)	exon-exon junction complex (GO:0035145)
spliceosomal snRNP complex (GO:0097525)	eukaryotic translation initiation factor 3 complex (GO:0005852)	nucleoplasm (GO:0005654)
chromosomal part (GO:0044427)	**extracellular region (GO:0005576)**	nucleoplasm part (GO:0044451)
Sm-like protein family complex (GO:0120114)	aminoacyl-tRNA synthetase multienzyme complex (GO:0017101)	nucleus (GO:0005634)
peptidase complex (GO:1905368)	catalytic step 2 spliceosome (GO:0071013)	catalytic complex (GO:1902494)
small nuclear ribonucleoprotein complex (GO:0030532)	mitochondrion (GO:0005739)	cell part (GO:0044464)
mitochondrial membrane (GO:0031966)	proteasome core complex (GO:0005839)	cell (GO:0005623)

Figure S5
