# Supplementary material for: A novel Microproteomic Approach Using Laser Capture Microdissection to Study Cellular Protrusions
Source: Int J Mol Sci. 2019 Mar 7;20(5):1172. doi: 10.3390/ijms20051172 (PMC6429397; doi:10.3390/ijms20051172)
Supplement: Supplementary file 1 [file ijms-20-01172-s001.zip › New-Fig S-4A-s.pdf]

A

hCAD PROTRUSIONS: TOTAL UNIQUE PROTEINS (642)									
2210010C04RIK	C1QBP	EEF1G	GNAS	KPNA4	PABPC1	PSMC1	RPLP0	SNRPD1	UQCRC1
4930519F16RIK	CACYBP	EEF2	GNB1	KPNB1	PABPC2	PSMC2	RPLP1	SNRPD3	UQCRC2
A1E2B8	CALCOCO1	EIF2S1	RACK1	KRT90	PABPN1	PSMC3	RPLP2	SNRPE	USO1
AARS	CALM1	EIF2S3X	GOT2	L1CAM	PAFAH1B2	PSMC4	RPN2	SNU13	VAC14
ABCE1	CAMK1G	EIF3A	GPI	LAP3	PAFAH1B3	PSMC5	RPS10	SNX3	VARS
ACAA1A	CAMK2D	EIF3B	GRK5	LDHA	PAICS	PSMC6	RPS11	SOD2	VAT1
ACAT1	CAND1	EIF3C	GRLF1	LIG1	PARP1	PSMD1	RPS12	SPATA7	VCL
ACLY	CANX	EIF3D	GRPEL1	LIN7C	PCBP1	PSMD11	RPS13	SPEN	VCP
ACO2	CAP1	EIF3E	GSDMA3	LMNA	PCBP2	PSMD12	RPS14	SPTAN1	VDAC1
ACOT7	CAPRIN1	EIF3F	GSTP1	LRPPRC	PCBP3	PSMD2	RPS15	SPTBN1	VDAC2
ACTB	CAPZA2	EIF3G	H2AFX	LRRC59	PCDHGC4	PSMD3	RPS15A	SRM	VDAC3
ACTG1	CARS	EIF3J2	H3F3A	LUC7L2	PCNA	PSMD5	RPS16	SRSF10	VGF
ACTN1	CCDC124	EIF3L	HADH	LYZ1	PDAP1	PSMD7	RPS17	SRSF3	VIM
ACTR1A	CCT2	EIF3M	HARS	MAP1B	PDCD5	PSME3	RPS18	SSB	VKORC1L1
ACTR1B	CCT3	EIF4A1	HDAC6	MAP1LC3B	PDCD6IP	PSPH	RPS19	SSR1	VMN2R66
ACTR2	CCT4	EIF4H	HIST1H1A	MARCKSL1	PDIA3	PTBP1	RPS2	SSR4	VPS35
ACTR3	CCT5	EIF5	HIST1H1B	MARS	PDIA6	PTPRD	RPS20	ST13	XPNPEP1
ADRM1	CCT6A	EIF5A	HIST1H1C	MAT2A	PDXP	PYM1	RPS21	STIP1	XPO5
AKAP12	CCT7	EIF6	HIST1H1E	MAT2B	PEBP1	Q505R5	RPS23	STMN1	YARS
AKR1B1	CCT8	ELAVL1	HIST1H2AF	MATR3	PES1	Q80Y73	RPS24	STMN2	YBX1
ALAD	CD47	ELAVL2	HIST1H2BB	MCM2	PFDN1	Q8VEQ0	RPS25	STOML2	YBX2
ALB	CDC42	ELAVL4	HIST1H2BF	MCM3	PFDN2	Q9CQE8	RPS26	SUPT6H	YBX3
ALDH9A1	CFL1	ELMO2	HIST1H2BP	MCM4	PFDN4	RAB11A	RPS27L	SYN2	YME1L1
ALDOA	CHCHD2	EMB	HIST1H3B	MCM5	PFDN5	RAB1A	RPS28	SYNCRIP	YWHAE
ANXA2	CHGB	ENO1	HIST1H4A	MCM6	PFDN6	RAB2A	RPS3	SYTL4	YWHAG
ANXA6	CHMP4B	EPRS	HIST2H2AA1	MCM7	PFN1	RAB5C	RPS3A	TAGLN2	YWHAH
AP1B1	CKAP4	ESCO1	HIST2H2AC	MDH1	PGAM1	RAB7A	RPS4X	TALDO1	YWHAQ
APRT	CLIC1	ESYT1	HMGB2	MDH2	PGD	RAB8B	RPS5	TARDBP	YWHAZ
ARF1	CLTC	ETF1	HNRNPA1	ME1	PGK1	RAC2	RPS6	TCP1	ZFP606
ARHGDIA	CNN3	EXOSC2	HNRNPA2B1	MEST	PGLS	RAN	RPS7	TENM2	ZPR1
ARL2	COPG1	EZR	HNRNPAB	METAP2	PHB	RANBP1	RPS8	TFRC	
ARL3	COPS3	FAM160A2	HNRNPC	MIF	PHB2	RANGAP1	RPS9	THOP1	
ARPC4	COPS7A	FAM171A2	HNRNPD	MSN	PHGDH	RAP1A	RPSA	TIMM13	
ARPC5	CORO1C	FAM49B	HNRNPF	MTCO2	PITPNA	RARS	RSU1	TIMM23	
ARVCF	COX5A	FARSA	HNRNPH1	MTHFD1	PKM	RBM3	RTN4	TIMM50	
ASIC2	CRIP2	FARSB	HNRNPH3	MTHFD1L	POLR2H	RBMX	RUVBL1	TKT	
ASL	CS	FASN	HNRNPK	MTHFD2	PPIA	RCC2	RUVBL2	TLN1	
ASNA1	CSDE1	FAU	HNRNPM	MYBBP1A	PPIB	RMDN3	SARNP	TLR12	
ASNS	CSE1L	FDPS	HNRNPR	MYH10	PPID	RNF181	SARS	TMCC2	
ASS1	CSNK2A1	FDXR	HNRNPU	MYH9	PPP1CA	RPA1	SCARB2	TMEM33	
ATIC	CYB5R3	FH	HSD17B10	MYL12B	PPP1CB	RPA3	SCRIB	TOMM22	
ATL2	DAZAP1	FIS1	HSP90AA1	MYL6	PPP2R1A	RPAP1	SDHA	TOMM34	
ATP1A1	DCTN2	FLNA	HSP90AB1	NAA25	PPP5C	RPL10A	SEPT11	TOP2A	
ATP1A3	DDOST	FLT4	HSP90B1	NACA	PRDX1	RPL10L	SERBP1	TPI1	
ATP5A1	DDX1	FMR1	HSPA1L	NAF1	PRDX2	RPL11	SET	TPM3	
ATP5B	DDX39B	FN1	HSPA4	NAP1L1	PRDX3	RPL12	SETX	TPPP3	
ATP5D	DDX3X	FSCN1	HSPA5	NARS	PRDX4	RPL13	SF3A3	TPR	
ATP5F1	DDX47	FTL1	HSPA8	NCAM1	PRDX6	RPL14	SF3B1	TRAP1	
ATP5H	DDX5	FUS	HSPA9	NCAPG	PREP	RPL15	SH3D21	TRIM28	
ATP5J2	DDX6	G3BP1	HSPD1	NCL	PRKACA	RPL18	SHANK2	TRIP13	
ATP5O	DERL1	G3BP2	HSPE1	NLRP4C	PRKACB	RPL22	SHMT2	TRMT112	
ATP6V0A1	DHX15	G6PDX	HSPH1	NME1	PRPH	RPL23	SIK3	TRY10	
ATP6V1A	DHX9	GAK	HYOU1	NMT1	PRPS1	RPL23A	SKINT5	TTN	
ATP6V1E1	DKC1	GANAB	IL1RAPL1	NOLC1	PRPSAP1	RPL24	SLC1A4	TUBA1A	
ATP6V1G1	DLAT	GAPDH	ILF2	NONO	PRPSAP2	RPL27	SLC1A5	TUBA1B	
ATP6V1H	DMXL1	GAPDHS	ILF3	NOP56	PSAT1	RPL27A	SLC25A10	TUBB2A	
ATXN10	DNMT3A	GARS	IPO5	NPM1	PSMA2	RPL30	SLC25A12	TUBB2B	
AU015228	DPY30	GCH1	IPO7	NPM3	PSMA3	RPL31	SLC25A3	TUBB3	
BAG6	DPYSL2	GDI2	IQGAP1	NQO1	PSMA4	RPL32-PS	SLC25A4	TUBB4B	
BANF1	DPYSL3	GFM1	ITGB1	NSFL1C	PSMA5	RPL34	SLC25A5	TUBB5	
BASP1	DSP	GJD3	JUP	NSUN2	PSMA6	RPL35	SLC2A3	TUFM	
BAX	DSTN	GLMN	KARS	NUDC	PSMA7	RPL38	SLC3A2	TXNRD1	
BAZ1B	DYNC1H1	GLRX3	KATNA1	NUP133	PSMB1	RPL4	SNAP23	UBA1	
BC002059	E2F4	GM10126	KATNAL2	OAT	PSMB2	RPL5	SNAP25	UBA52	
BC094435	EEF1A1	GM10610	KCNAB2	OGDH	PSMB3	RPL6	SND1	UBAP2L	
BRE	EEF1A2	GM5409	KDELC2	OLA1	PSMB5	RPL7	SNRNP200	UBE2D3	
BTF3	EEF1B	GM5771	KIF4	P4HB	PSMB6	RPL7A	SNRPA	UBQLN2	
BZW2	EEF1D	GM6583	KPNA2	PA2G4	PSMB7	RPL9	SNRPB	UMPS	

hCAD PROTRUSIONS: "IN 2" (190)		
AARS	HSPA4	RPLP2
ABCE1	HSPA5	RPS10
ACAT1	HSPA8	RPS12
ACLY	HSPA9	RPS13
ACTB	HSPD1	RPS16
ACTG1	IL1RAPL1	RPS17
ACTN1	KARS	RPS18
ADRM1	KATNAL2	RPS19
AKR1B1	KPNA2	RPS2
ALB	KPNB1	RPS23
ALDOA	LDHA	RPS24
ANXA2	MAP1B	RPS25
APRT	MAT2B	RPS28
ARPC4	MDH1	RPS3
ASL	MDH2	RPS3A
ASNS	MYL6	RPS4X
ATP5A1	NACA	RPS5
ATP5B	NCAM1	RPS7
ATP6V1A	NCL	RPS8
BTF3	NME1	RPS9
C1QBP	NPM1	RPSA
CAPRIN1	NSFL1C	SERBP1
CAPZA2	OLA1	SET
CCT2	PCBP2	SHMT2
CCT3	PCBP3	SLC25A3
CCT4	PDAP1	SLC25A5
CCT5	PDIA3	SLC3A2
CCT6A	PDIA6	SNRPD3
CCT8	PFDN2	SPEN
CFL1	PFN1	SPTAN1
CLTC	PGAM1	SRM
CORO1C	PHB2	ST13
DDX39B	PKM	STIP1
DDX3X	PPIA	SYNCRIP
DPYSL3	PPP1CA	TAGLN2
DSP	PRDX1	TCP1
EEF1A1	PRDX2	TPI1
EEF1A2	PRDX4	TRAP1
EEF1D	PRKACA	TUBA1A
EEF1G	PRPH	TUBA1B
EEF2	PSAT1	TUBB3
EIF2S1	PSMA5	TUBB4B
EIF3B	PSMA6	TUBB5
EIF4A1	PSMA7	UBA1
EIF4H	PTBP1	UBA52
ELAVL1	RAB7A	UQCRC2
EMB	RAN	VCP
ENO1	RANBP1	VDAC1
ETF1	RAP1A	VDAC3
FASN	RBM3	VGF
FLNA	RBMX	VIM
FN1	RPAP1	YWHAE
FSCN1	RPL10A	YWHAG
G3BP1	RPL11	YWHAZ
GAPDH	RPL12	
GDI2	RPL13	
GOT2	RPL14	
HIST1H1A	RPL18	
HIST1H2AF	RPL23	
HIST1H4A	RPL27A	
HNRNPA2B1	RPL30	
HNRNPAB	RPL31	
HNRNPD	RPL35	
HNRNPK	RPL4	
HNRNPU	RPL5	
HSP90AA1	RPL7	
HSP90AB1	RPL9	
HSP90B1	RPLP0	

hCAD PROTRUSIONS: "exclusive" (37)
ARVCF
ASIC2
ATP1A3
AU015228
BC002059
CAMK1G
DMXL1
E2F4
EIF3J2
ESCO1
FAM160A2
FAM171A2
FLT4
GAPDHS
GM10126
GM10610
GM6583
GRK5
GRLF1
GSDMA3
HIST1H2BP
KATNA1
KDELC2
PABPC2
PTPRD
Q505R5
Q8VEQ0
RAB8B
RAC2
RPL32-PS
SHANK2
SKINT5
TENM2
TLR12
TMCC2
YBX2
ZFP606

Figure S4
