# Supplementary material for: A novel Microproteomic Approach Using Laser Capture Microdissection to Study Cellular Protrusions
Source: Int J Mol Sci. 2019 Mar 7;20(5):1172. doi: 10.3390/ijms20051172 (PMC6429397; doi:10.3390/ijms20051172)
Supplement: Supplementary file 1 [file ijms-20-01172-s001.zip › New-Fig S6A-s.pdf]

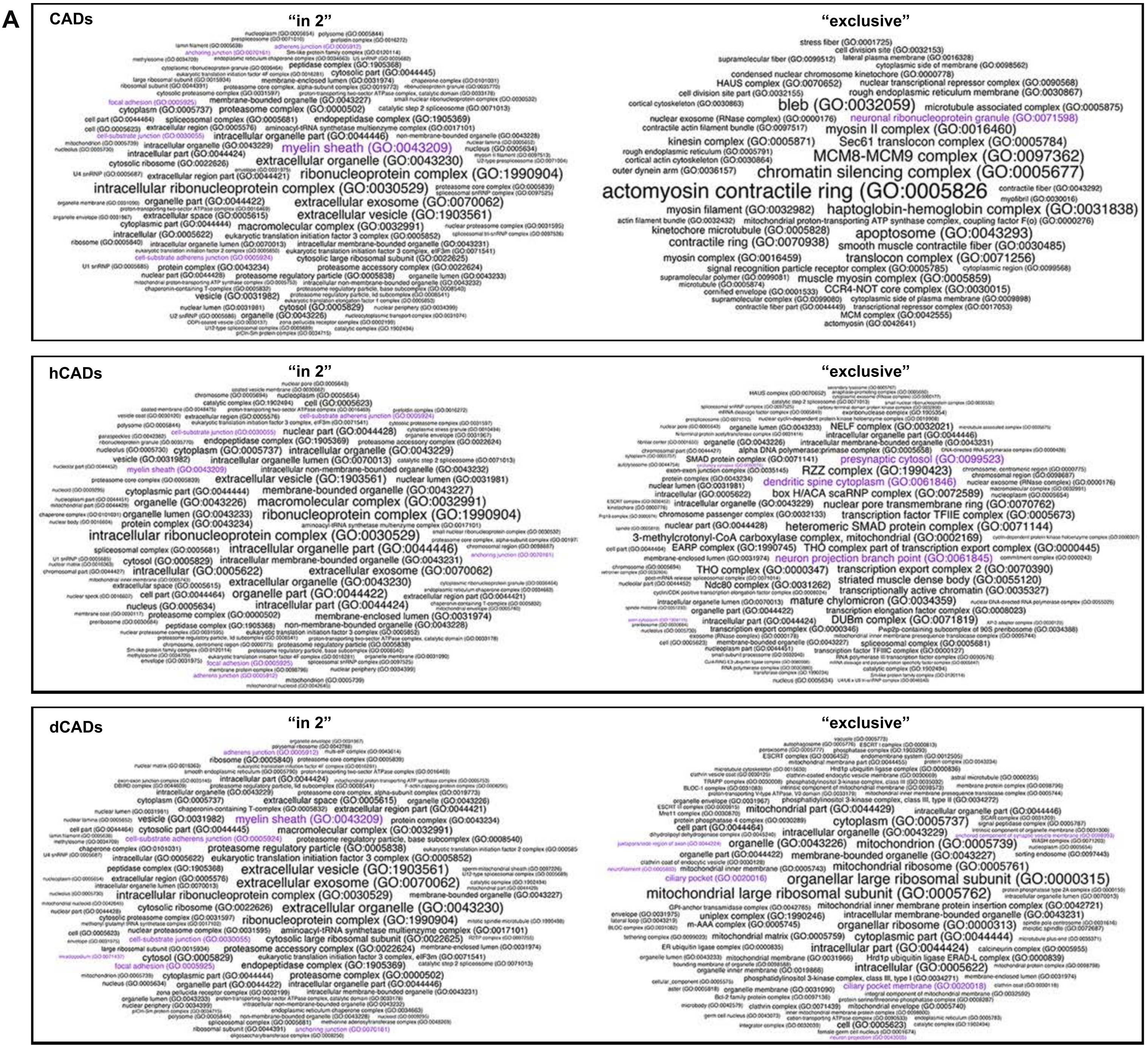
A
CADs
"in 2"
"exclusive"
myelin sheath (GO:0043209)
extracellular organelle (GO:0043230)
ribonucleoprotein complex (GO:1990904)
intracellular ribonucleoprotein complex (GO:0030529)
extracellular exosome (GO:0070062)
extracellular vesicle (GO:1903561)
bleb (GO:0032059)
neuronal ribonucleoprotein granule (GO:0071598)
myosin II complex (GO:0016460)
Sec61 translocon complex (GO:0005784)
MCM8-MCM9 complex (GO:0097362)
chromatin silencing complex (GO:0005677)
actomyosin contractile ring (GO:0005826)
haptoglobin-hemoglobin complex (GO:0031838)
apoptosome (GO:0043293)
translocon complex (GO:0071256)
hCADs
"in 2"
"exclusive"
macromolecular complex (GO:0032991)
ribonucleoprotein complex (GO:1990904)
intracellular ribonucleoprotein complex (GO:0030529)
intracellular organelle part (GO:0044446)
extracellular exosome (GO:0070062)
organelle part (GO:0044422)
presynaptic cytosol (GO:0099523)
RZZ complex (GO:1990423)
dendritic spine cytoplasm (GO:0061846)
box H/ACA scaRNP complex (GO:0072589)
transcription factor TFIIE complex (GO:0005673)
heteromeric SMAD protein complex (GO:0071144)
3-methylcrotonyl-CoA carboxylase complex, mitochondrial (GO:0002169)
neuron projection branch point (GO:0061845)
dCADs
"in 2"
"exclusive"
myelin sheath (GO:0043209)
extracellular vesicle (GO:1903561)
extracellular exosome (GO:0070062)
intracellular ribonucleoprotein complex (GO:0030529)
extracellular organelle (GO:0043230)
ribonucleoprotein complex (GO:1990904)
cytoplasm (GO:0005737)
mitochondrion (GO:0005739)
organellar large ribosomal subunit (GO:0000315)
mitochondrial large ribosomal subunit (GO:0005762)
organellar ribosome (GO:0000313)
ciliary pocket (GO:0020016)
intracellular part (GO:0044424)
intracellular (GO:0005622)

Figure S6
