# Supplementary material for: A novel Microproteomic Approach Using Laser Capture Microdissection to Study Cellular Protrusions
Source: Int J Mol Sci. 2019 Mar 7;20(5):1172. doi: 10.3390/ijms20051172 (PMC6429397; doi:10.3390/ijms20051172)
Supplement: Supplementary file 1 [file ijms-20-01172-s001.zip › New-Fig S-4C-with legend-s.pdf]

C

GC: TOTAL UNIQUE PROTEINS (444)						
4930519F16RIK	CS	GPI	MAP1LC3B	PSMA3	RPS17	TUBA1C
A0A097PUD0	CTNNA1	GSTP1	MARCKSL1	PSMA5	RPS18	TUBB3
A0A097PUG4	D1PAS1	H2AFV	MAT2A	PSMA6	RPS19	TUBB4A
ACAT1	DAP	H2AFX	MAT2B	PSMA7	RPS2	TUBB4B
ACLY	DAPK1	HARS	MBP	PSMB1	RPS20	TUBB5
ACOT7	DDOST	HBB-B1	MCM3	PSMB3	RPS21	TXN
ACTA1	DDX21	HDAC7	MDH1	PSMB6	RPS23	UBA1
ACTBL2	DDX39B	HGH1	MDH2	PSMB7	RPS24	UBA52
ACTB	DDX3X	HINT1	METTL3	PSMD12	RPS25	UBE2J1
ACTG1	DDX5	HIST1H1A	MIF	PSMD4	RPS26	UBE2N
ACTN1	DHFR	HIST1H1C	MROH6	PSME3	RPS28	UBE2V1
AKR1B1	DIP2C	HIST1H1D	MYBBP1A	PSPH	RPS29	UCHL1
ALB	DNAH5	HIST1H1E	MYG1	PTBP1	RPS3	ULK2
ALDOA	DPYSL3	HIST1H2AB	MYH9	PTMA	RPS3A	UQCRC1
ANXA2	DSG1B	HIST1H2BH	MYL12A	PTPRZ1	RPS4X	UQCRFS1
APRT	DSP	HIST1H2BM	MYL12B	Q80Y73	RPS5	USP5
ARF1	DSTN	HIST1H3A	MYL6	DSCAML1	RPS7	UTP14B
ARF4OS	EEF1A1	HIST1H3B	NAA25	RAB11B	RPS8	VAT1
ARG1	EEF1B	HIST1H4A	NACA	RAB1A	RPS9	VCP
ARHGDIA	EEF1D	HIST2H2AC	NAP1L1	RAB1B	RPSA	VCPIP1
ARL3	EEF1G	HIST2H2BB	NCAM1	RAB2A	RWDD4	VDAC1
ARPC4	EEF2	HIST3H2BB	NCL	RAB39A	S100A14	VDAC3
ASL	EIF2A	HMGN1	NME1	RAB7A	S100A6	VIM
ASNA1	EIF2S1	HMGN2	NMT1	RACK1	SARNP	VPS13B
ASNS	EIF2S3X	HNRNPA0	NPM1	RALGAPA2	SARS	VPS39
ASS1	EIF3F	HNRNPA1	NSUN2	RAN	SCN2A	XPNPEP1
ATAT1	EIF3G	HNRNPA2B1	NUDC	RAP1A	SDHA	YBX1
ATP5A1	EIF3L	HNRNPAB	OLA1	RARS	SEPT2	YBX3
ATP5B	EIF4A1	HNRNPD	P4HB	RBM3	SERBP1	YWHAB
ATP5F1	EIF4B	HNRNPF	PA2G4	RBMX	SERPINC1	YWHAE
ATP5O	EIF4H	HNRNPH1	PABPC1	RNF181	SFN	YWHAG
ATP6V1B2	EIF5A	HNRNPH3	PAFAH1B3	RPA3	SLC25A3	YWHAQ
B7U9H2	ELAVL1	HNRNPK	PCBP1	RPAP1	SLC25A4	YWHAZ
BASP1	ELAVL4	HNRNPM	PCBP2	RPGRIP1L	SLC25A5	ZBED4
BC094435	ENO1	HNRNPU	PCBP3	RPL10A	SLC3A2	ZFP65
BLVRB	ENO3	HSP90AA1	PDAP1	RPL10L	SNCG	ZSCAN20
BPIFC	EPRS	HSP90AB1	PDCD5	RPL11	SND1	
BTF3	ETF1	HSP90B1	PDE4D	RPL12	SNRPA	
BZW2	EZR	HSPA4	PDHB	RPL13	SNRPD3	
CACYBP	FABP5	HSPA5	PDIA3	RPL14	SRM	
CAD	FAM234A	HSPA8	PDIA6	RPL17	SRSF3	
CALM1	FASN	HSPA9	PEBP1	RPL18	ST13	
CALU	FAU	HSPD1	PFDN2	RPL22	STIP1	
CAND1	FBXO44	HSPE1	PFDN4	RPL23	STMN1	
CAPRIN1	FDPS	HSPH1	PFKL	RPL23A	STOML2	
CAPZA2	FGF22	HYOU1	PFN1	RPL24	SYN2	
CCDC124	FH	IL1RAPL1	PGAM1	RPL26	SYNCRIP	
CCDC93	FIS1	ILF2	PGD	RPL27	SYTL4	
CCT2	FLNA	ITGB1	PGK1	RPL31	TAGLN2	
CCT3	FLNB	ITIH2	PGLS	RPL34	TARDBP	
CCT4	FN1	JUP	PHB	RPL35	TARS	
CCT5	FSCN1	KARS	PHB2	RPL38	TBCA	
CCT6A	FUBP1	KCNAB2	PHGDH	RPL4	TCP1	
CCT7	FXR1	KRT90	PKM	RPL5	TF	
CCT8	G3BP1	KIF20B	PLEC	RPL6	TFRC	
CDC42	GANAB	KIF4	POU2F1	RPL7	TIAL1	
CDV3	GAPDH	KPNA2	PPIA	RPL7A	TKT	
CFL1	GARS	KPNB1	PPL	RPL9	TMPO	
CHCHD2	GCLM	KXD1	PRDM16	RPLP0	TPI1	
CHMP4B	GDI2	LDHA	PRDX1	RPLP1	TPM2	
CLCF1	GLRX3	LF1	PRDX2	RPLP2	TPM3	
CLDN3	GM10334	LGALS1	PRDX4	RPS10	TPPP3	
CLIC1	GM13125	LOXHD1	PREP	RPS11	TPT1	
CLTC	GM19638	LRMP	PRKACA	RPS12	TRAJ3	
COBL	GM5409	LRRC47	PRKACB	RPS13	TRAP1	
COL6A1	GM5414	LRRC59	PRPH	RPS14	TRY10	
CORO1C	GNA12	LYZ1	PSAT1	RPS15A	TUBA1A	
CRMP1	GNAS	MAP1B	PSMA2	RPS16	TUBA1B	

GC: "in 2" (193)		
ACLY	HSPA5	RPL4
ACTB	HSPA8	RPL6
ACTG1	HSPA9	RPL7A
ACTN1	HSPD1	RPL9
AKR1B1	HSPE1	RPLP0
ALB	HYOU1	RPLP1
ALDOA	IL1RAPL1	RPLP2
ANXA2	ILF2	RPS10
APRT	JUP	RPS12
ARHGDIA	KIF20B	RPS14
ASL	KPNA2	RPS15A
ASNA1	KPNB1	RPS18
ASNS	LDHA	RPS19
ATP5A1	LGALS1	RPS2
ATP5B	LRRC47	RPS24
BPIFC	MDH1	RPS25
CACYBP	MDH2	RPS26
CCT2	MROH6	RPS28
CCT3	MYL12A	RPS3
CCT4	NACA	RPS3A
CCT5	NAP1L1	RPS4X
CCT6A	NCL	RPS5
CCT7	NPM1	RPS7
CCT8	NUDC	RPS8
CFL1	P4HB	RPS9
CLDN3	PA2G4	RPSA
DDX39B	PABPC1	SARNP
DDX5	PCBP1	SFN
DPYSL3	PCBP2	SLC25A4
DSP	PDCD5	SLC25A5
EEF1A1	PDE4D	SLC3A2
EEF1D	PDIA3	SRSF3
EEF1G	PDIA6	STMN1
EEF2	PEBP1	SYNCRIP
EIF4A1	PFDN2	SYTL4
EIF5A	PFDN4	TAGLN2
ELAVL1	PFN1	TARDBP
ENO1	PGK1	TARS
ETF1	PHB	TCP1
FAU	PHGDH	TPI1
FDPS	PKM	TPM3
FH	PPIA	TRAP1
FLNA	PRDX1	TUBA1A
FLNB	PRDX2	TUBA1C
FN1	PRDX4	TUBB3
FSCN1	PRPH	TUBB5
G3BP1	PSMA5	UBA1
GAPDH	PSMA7	UBA52
GARS	PSMB6	VAT1
GDI2	PTBP1	VCP
GLRX3	RAB1A	VCPIP1
GSTP1	RACK1	VDAC1
HINT1	RALGAPA2	VIM
HIST1H2AB	RAN	XPNPEP1
HIST1H3A	RAP1A	YBX1
HIST1H4A	RBM3	YWHAE
HIST2H2AC	RBMX	YWHAZ
HNRNPA1	RPAP1	
HNRNPA2B1	RPL10A	
HNRNPAB	RPL11	
HNRNPD	RPL12	
HNRNPF	RPL14	
HNRNPK	RPL18	
HNRNPU	RPL22	
HSP90AA1	RPL23A	
HSP90AB1	RPL24	
HSP90B1	RPL31	
HSPA4	RPL35	

GC: Exclusive (39)
ACTA1
ARF4OS
ATAT1
B7U9H2
BLVRB
BPIFC
CCDC93
COBL
COL6A1
D1PAS1
DAP
DAPK1
DIP2C
DNAH5
DSG1B
ENO3
FAM234A
FBXO44
GM10334
GM13125
GM19638
GNA12
HDAC7
KRT90
KXD1
LF1
LOXHD1
PPL
PTPRZ1
RAB39A
RPGRIP1L
SCN2A
TPM2
UBE2J1
ULK2
VPS13B
VPS39
ZBED4
ZSCAN20

Figure S4: Comparison of protein lists obtained by LCM/MS, identified with different analysis approaches. Comparison of the “Total unique protein” lists vs protein lists obtained from proteins “in at least 2 samples” (additive approach) or “exclusive” protein lists (subtractive approach) obtained from LCM/MS of (A) hCAD protrusions; (B) dCAD protrusions; or (C) GCs
