# Supplementary material for: A novel Microproteomic Approach Using Laser Capture Microdissection to Study Cellular Protrusions
Source: Int J Mol Sci. 2019 Mar 7;20(5):1172. doi: 10.3390/ijms20051172 (PMC6429397; doi:10.3390/ijms20051172)
Supplement: Supplementary file 1 [file ijms-20-01172-s001.zip › New-Fig S5H-s.pdf]

H

GO Terms Related to Protrusions	GO Terms Related to Protrusions	GO Terms Related to Protrusions
9+0 non-motile cilium (GO:0097731)	dendritic spine membrane (GO:0032591)	non-motile cilium (GO:0097730)
9+2 motile cilium (GO:0097729)	dendritic spine neck (GO:0044326)	paranodal junction (GO:0033010)
actin-based cell projection (GO:0098858)	dendritic tree (GO:0097447)	paranode region of axon (GO:0033270)
adherens junction (GO:0005912)	excitatory synapse (GO:0060076)	periciliary membrane compartment (GO:1990075)
amyloid-beta complex (GO:0106003)	fascia adherens (GO:0005916)	perikaryon (GO:0043204)
anchored component of synaptic vesicle membrane (GO:0098993)	filopodium (GO:0030175)	perineuronal net (GO:0072534)
anchoring junction (GO:0070161)	filopodium membrane (GO:0031527)	peripheral region of growth cone (GO:0090725)
apical dendrite (GO:0097440)	filopodium tip (GO:0032433)	photoreceptor cell cilium (GO:0097733)
apical ectoplasmic specialization (GO:0061831)	flotillin complex (GO:0016600)	photoreceptor connecting cilium (GO:0032391)
apical junction complex (GO:0043296)	focal adhesion (GO:0005925)	photoreceptor inner segment (GO:0001917)
apical tubulobulbar complex (GO:0061828)	gap junction (GO:0005921)	photoreceptor outer segment membrane (GO:0042622)
astrocyte end-foot (GO:0097450)	glial cell projection (GO:0097386)	pinceau fiber (GO:1990031)
astrocyte projection (GO:0097449)	glial limiting end-foot (GO:0097451)	pinosome (GO:0044352)
asymmetric synapse (GO:0032279)	growth cone (GO:0030426)	plasma membrane bounded cell projection (GO:0120025)
axon (GO:0030424)	growth cone filopodium (GO:1990812)	plasma membrane bounded cell projection cytoplasm (GO:0032838)
axon cytoplasm (GO:1904115)	growth cone membrane (GO:0032584)	plasma membrane bounded cell projection part (GO:0120038)
axon hillock (GO:0043203)	growth cone part (GO:0090723)	plasma membrane protein complex (GO:0098797)
axon initial segment (GO:0043194)	hemidesmosome (GO:0030056)	plasma membrane raft (GO:0044853)
axon part (GO:0033267)	immunological synapse (GO:0001772)	postsynapse (GO:0098794)
axon terminus (GO:0043679)	inner dynein arm (GO:0036156)	postsynaptic actin cytoskeleton (GO:0098871)
axonal growth cone (GO:0044295)	integral component of postsynaptic density membrane (GO:0099061)	postsynaptic cytoskeleton (GO:0099571)
axonal spine (GO:0044308)	integral component of postsynaptic specialization membrane (GO:0099060)	postsynaptic cytosol (GO:0099524)
axonemal dynein complex (GO:0005858)	integral component of synaptic vesicle membrane (GO:0030285)	postsynaptic density (GO:0014069)
axonemal microtubule (GO:0005879)	intercellular bridge (GO:0045171)	postsynaptic density membrane (GO:0098839)
axoneme (GO:0005930)	internode region of axon (GO:0033269)	postsynaptic early endosome (GO:0098842)
axoneme part (GO:0044447)	intrinsic component of postsynaptic density membrane (GO:0099146)	postsynaptic endosome (GO:0098845)
basal ectoplasmic specialization (GO:0061832)	intrinsic component of postsynaptic specialization membrane (GO:0098948)	postsynaptic membrane (GO:0045211)
brush border (GO:0005903)	intrinsic component of synaptic vesicle membrane (GO:0098563)	postsynaptic recycling endosome (GO:0098837)
brush border membrane (GO:0031526)	invadopodium (GO:0071437)	postsynaptic specialization (GO:0099572)
caveola (GO:0005901)	invadopodium membrane (GO:0071438)	postsynaptic specialization membrane (GO:0099634)
cell projection (GO:0042995)	juxtaparanode region of axon (GO:0044224)	presynapse (GO:0098793)
cell projection membrane (GO:0031253)	lamellipodium (GO:0030027)	presynaptic active zone (GO:0048786)
cell projection part (GO:0044463)	lamellipodium membrane (GO:0031258)	presynaptic active zone membrane (GO:0048787)
cell surface furrow (GO:0097610)	leading edge of lamellipodium (GO:0061851)	presynaptic cytosol (GO:0099523)
cell trailing edge (GO:0031254)	lipopolysaccharide receptor complex (GO:0046696)	presynaptic membrane (GO:0042734)
cell-cell adherens junction (GO:0005913)	macropinosome (GO:0044354)	proximal dendrite (GO:1990635)
cell-cell contact zone (GO:0044291)	macropinocytic cup (GO:0070685)	pseudopodium (GO:0031143)
cell-substrate adherens junction (GO:0005924)	main axon (GO:0044304)	ruffle (GO:0001726)
cell-substrate junction (GO:0030055)	membrane microdomain (GO:0098857)	ruffle membrane (GO:0032587)
central region of growth cone (GO:0090724)	membrane raft (GO:0045121)	Schmidt-Lanterman incisure (GO:0043220)
ciliary basal body (GO:0036064)	microspike (GO:0044393)	Schwann cell microvillus (GO:0097454)
ciliary base (GO:0097546)	microvillus (GO:0005902)	site of polarized growth (GO:0030427)
ciliary membrane (GO:0060170)	microvillus membrane (GO:0031528)	somatodendritic compartment (GO:0036477)
ciliary part (GO:0044441)	motile cilium (GO:0031514)	sperm annulus (GO:0097227)
ciliary plasm (GO:0097014)	myelin sheath (GO:0043209)	sperm flagellum (GO:0036126)
ciliary rootlet (GO:0035253)	myelin sheath abaxonal region (GO:0035748)	spine apparatus (GO:0097444)
ciliary transition fiber (GO:0097539)	myelin sheath adaxonal region (GO:0035749)	stereocilia ankle link (GO:0002141)
ciliary transition zone (GO:0035869)	neurofibrillary tangle (GO:0097418)	stereocilia ankle link complex (GO:0002142)
cilium (GO:0005929)	neurofilament (GO:0005883)	stereocilia coupling link (GO:0002139)
cleavage furrow (GO:0032154)	neuromuscular junction (GO:0031594)	stereocilium (GO:0032420)
cluster of actin-based cell projections (GO:0098862)	neuron part (GO:0097458)	symmetric synapse (GO:0032280)
compact myelin (GO:0043218)	neuron projection (GO:0043005)	synapse (GO:0045202)
dendrite (GO:0030425)	neuron projection branch point (GO:0061845)	synapse part (GO:0044456)
dendrite cytoplasm (GO:0032839)	neuron projection cytoplasm (GO:0120111)	synaptic membrane (GO:0097060)
dendrite terminus (GO:0044292)	neuron projection membrane (GO:0032589)	synaptic vesicle (GO:0008021)
dendritic branch (GO:0044307)	neuron projection terminus (GO:0044306)	synaptic vesicle membrane (GO:0030672)
dendritic filopodium (GO:1902737)	neuron spine (GO:0044309)	terminal bouton (GO:0043195)
dendritic growth cone (GO:0044294)	neuron to neuron synapse (GO:0098984)	unconventional myosin complex (GO:0016461)
dendritic shaft (GO:0043198)	neuronal cell body (GO:0043025)	uropod (GO:0001931)
dendritic spine (GO:0043197)	neuronal cell body membrane (GO:0032809)	VCP-NSFL1C complex (GO:1990730)
dendritic spine cytoplasm (GO:0061846)	neuronal ribonucleoprotein granule (GO:0071598)	zonula adherens (GO:0005915)
dendritic spine head (GO:0044327)	node of Ranvier (GO:0033268)	

Figure S5: Comparison of top 50 GO Terms obtained by LCM/MS, identified with different analysis approaches. Top 50 GO Term lists from Total unique protein (left column); Proteins obtained from proteins “in at least 2 samples” (additive approach) (middle column); and “exclusive” proteins (subtractive approach) (right column) are shown for LCM/MS of (A) CADs; (B) hCADs; (C) dCADs; (D) hCAD protrusions; (E) dCAD protrusions; and (F) GCs. (G) Top 50 GO Term lists from Total unique protein (left column) and “exclusive” proteins from TNTs (subtractive approach) (right column). For all cases, GO Terms related to protrusions (violet), membrane (blue) and cytoskeleton (red) are highlighted. (H) The GO term list “related to protrusions”, highlighted in violet in each tables, is shown.
