# Supplementary material for: A novel Microproteomic Approach Using Laser Capture Microdissection to Study Cellular Protrusions
Source: Int J Mol Sci. 2019 Mar 7;20(5):1172. doi: 10.3390/ijms20051172 (PMC6429397; doi:10.3390/ijms20051172)
Supplement: Supplementary file 1 [file ijms-20-01172-s001.zip › New-Fig S-4B-s.pdf]

B

dCAD PROTRUSIONS: TOTAL UNIQUE PROTEINS (756)											
4930550L24RIK	ATIC	CHCHD3	EIF3B	GRPEL1	LAMTOR1	P4HB	PRKCSH	RPL13A	SEPT7	TLN1	YKT6
A0A097PUG4	ATL3	CHGB	EIF3F	GSN	LAP3	PA2G4	PRMT1	RPL14	SEPT9	TMEM120A	YWHAB
A0A097PUH2	ATP1A1	CHMP4B	EIF3L	GSR	LAPTM5	PABPC1	PRNP	RPL18	SERBP1	TMEM33	YWHAE
A2M	ATP1B1	CLIC1	EIF4A1	GSTM1	LASP1	PABPN1	PRPF19	RPL22	SERPINC1	TMX1	YWHAG
AARS	ATP2A2	CLPP	EIF4A2	GSTP1	LDHA	PAFAH1B1	PRPH	RPL23	SERPINF1	TMX4	YWHAH
ABCE1	ATP2B1	CLPTM1	EIF4B	H2AFJ	LGALS1	PAFAH1B2	PRR27	RPL23A	SETX	TNPO1	YWHAQ
ACAA2	ATP5A1	CLTC	EIF4H	H3F3C	LIN7C	PAFAH1B3	PSAT1	RPL3	SF3B3	TOMM70A	YWHAZ
ACAT1	ATP5B	CNBP	ELAVL1	HADH	LNP	PAICS	PSMA1	RPL30	SH3D21	TPD52	ZYX
ACAT2	ATP5C1	COPG1	EMB	HARS	LONP1	PALLD	PSMA2	RPL35	SH3GLB2	TPI1	
ACLY	ATP5D	COPS3	EMD	HBB-Y	LRPPRC	PARK7	PSMA3	RPL38	SHMT2	TPM1	
ACO2	ATP5E	COPS4	ENAH	HGFAC	LRRC40	PC	PSMA4	RPL4	SIK3	TPM3	
ACOT2	ATP5F1	CORO1C	ENO1	HINT1	LRRC47	PCBP1	PSMA5	RPL5	SLC12A9	TPM4	
ACOT7	ATP5H	COX4I1	EPHX1	HIST1H2AF	LRRC59	PCBP2	PSMA6	RPL6	SLC16A1	TPPP3	
ACTB	ATP5J2	COX5A	EPRS	HIST1H2BB	LSS	PCBP3	PSMA7	RPL7	SLC1A5	TRAP1	
ACTG1	ATP5L	COX6C	ERLIN2	HIST1H2BF	LTA4H	PCDHGC4	PSMB1	RPL7A	SLC25A1	TRDMT1	
ACTN1	ATP5O	CPNE1	ERMP1	HIST1H4A	LYZ1	PCK2	PSMB2	RPL8	SLC25A11	TRMT112	
ACTN4	ATP6V0A1	CPT1A	ERP29	HK1	MANF	PCMT1	PSMB3	RPL9	SLC25A12	TRPV2	
ACTR1A	ATP6V0D1	CRIP2	ESYT1	HMGB2	MAP1A	PDAP1	PSMB4	RPLP0	SLC25A3	TRY10	
ACTR1B	ATP6V1A	CRMP1	ETF1	HMGN2	MAP1B	PDCD5	PSMB5	RPLP1	SLC25A4	TSTA3	
ACTR2	ATP6V1B2	CS	ETFA	HMOX2	MAP1LC3B	PDCD6IP	PSMB6	RPLP2	SLC25A5	TUBA1A	
ACTR3	ATP6V1C1	CSNK2B	ETFB	HNRNPA1	MAP1S	PDHB	PSMC1	RPN1	SLC27A1	TUBA1B	
ACTR3B	ATP6V1D	CSTB	EXOC8	HNRNPA2B1	MAP4	PDIA3	PSMC2	RPN2	SLC2A3	TUBA8	
ADPGK	ATP6V1E1	CTSD	FAM129A	HNRNPAB	MAPK1	PDIA4	PSMC6	RPS10	SLC39A7	TUBB2A	
ADSL	ATP6V1G1	CTTN	FARSA	HNRNPD	MAPK3	PDIA6	PSMD1	RPS11	SLC3A2	TUBB2B	
AFDN	ATP6V1H	CYB5B	FASN	HNRNPK	MAPRE1	PDXP	PSMD11	RPS12	SNAP25	TUBB3	
AFM	ATPIF1	CYB5R1	FASTKD2	HPCA	MARCKSL1	PEBP1	PSMD12	RPS13	SNCG	TUBB4B	
AHCY	ATXN10	CYB5R3	FDXR	HSD17B10	MAT2A	PEPD	PSMD14	RPS14	SND1	TUBB5	
AHCYL1	BAG3	CYC1	FH	HSD17B12	MAT2B	PFDN1	PSMD2	RPS15A	SNRPD1	TUBB6	
AHNAK	BASP1	CYCS	FIS1	HSD17B7	MBLAC2	PFDN2	PSMD4	RPS16	SNRPD3	TUFM	
AHSG	BAX	D10JHU81E	FKBP1A	HSP90AA1	MDH1	PFDN6	PSMD7	RPS17	SNX2	TXNDC5	
AIP	BRK1	DAD1	FKBP2	HSP90AB1	MDH2	PFKL	PSPH	RPS18	SNX25	UBA1	
AK2	BSG	DARS	FKBP4	HSP90B1	ME1	PFKP	PTK7	RPS19	SNX3	UBA52	
AKAP12	BZW2	DBI	FKBP8	HSPA1B	MIF	PFN1	PTRH2	RPS2	SNX5	UBE2D3	
AKR1B1	C1QBP	DBNL	FLG2	HSPA4	MROH6	PGAM1	PYCR2	RPS20	SOD1	UBE2N	
ALB	C3	DCAKD	FLNA	HSPA5	MSN	PGD	PYCRL	RPS21	SOD2	UBE2V1	
ALDH18A1	C4B	DCLK1	FLNB	HSPA8	MTCO2	PGK1	QDPR	RPS23	SPATA7	UBXN1	
ALDH1L2	CACYBP	DCTN2	FLNC	HSPA9	MTHFD1L	PGLS	RAB10	RPS24	SPR	UCHL1	
ALDH2	CAD	DDOST	FN1	HSPD1	MTHFD2	PGRMC1	RAB11B	RPS25	SPRY1	UGGT1	
ALDH9A1	CALCOCO1	DDX39B	FSCN1	HSPE1	MYDGF	PGRMC2	RAB14	RPS27L	SPTAN1	UQCRB	
ALDOA	CALM1	DDX3X	G6PDX	HSPH1	MYG1	PHB	RAB1A	RPS28	SPTBN1	UQCRC1	
ALDOC	CALR	DECR1	GAK	HYOU1	MYH10	PHB2	RAB1B	RPS29	SRM	UQCRC2	
ALG2	CALU	DERL1	GALK1	IARS	MYH14	PHGDH	RAB21	RPS3	SRP14	UQCRQ	
ANXA2	CAMK2D	DHRS1	GANAB	IDH2	MYH9	PICALM	RAB2A	RPS3A	SSR4	USP5	
ANXA4	CAND1	DLAT	GAP43	IDH3A	MYL12B	PIN4	RAB5A	RPS4X	ST13	USP9X	
ANXA5	CANX	DLD	GAPDH	IDH3B	MYL6	PKM	RAB5C	RPS5	STIP1	VAMP2	
ANXA6	CAP1	DLST	GARS	IL1RAPL1	NACA	PLEC	RAB6A	RPS6	STMN1	VAMP3	
AP2A2	CAPZA1	DNAJA1	GBA	IMPA1	NAF1	PLIN3	RAB7A	RPS7	STMN2	VAPB	
AP2B1	CAPZA2	DNAJC11	GCLM	INA	NAP1L1	PLOD3	RAC1	RPS8	STOM	VARS	
AP2M1	CAPZB	DNPEP	GDI1	INS1	NAPA	PMM2	RACK1	RPS9	STOML2	VAT1	
APMAP	CARS	DPYSL2	GDI2	IQGAP1	NARS	PMVK	RAN	RPSA	STRAP	VAT1L	
APRT	CAT	DPYSL3	GJD3	ISOC1	NCAM1	POR	RANBP1	RTN4	STRN3	VBP1	
ARF1	CBR1	DPYSL5	GLIPR2	ITGB1	NDUFA4	PPIA	RAP1B	RUVBL1	STX12	VCP	
ARF4	CCDC115	DRG2	GLUD1	ITIH1	NEFH	PPIB	RARS	S100A11	STX7	VCPIP1	
ARF5	CCDC124	DSP	GM11214	ITIH2	NLE1	PPID	RBM14	S100A6	STXBP1	VDAC1	
ARG1	CCR9	DSTN	GM11639	ITIH3	NME1	PPP1CA	RBM3	SACM1L	SUCLG1	VDAC2	
ARHGDIA	CCT2	DYNC1H1	GM15013	ITIH4	NME2	PPP1CB	RCN2	SAR1A	SUCLG2	VDAC3	
ARL3	CCT3	DYNLT1	GM20425	JUP	NMT1	PPP2R1A	RDH11	SARNP	SYN1	VGF	
ARL6IP5	CCT4	ECH1	GM43738	KATNAL2	NPTN	PPP2R2A	RDX	SARS	SYNCRIP	VIM	
ARL8A	CCT5	ECHS1	GM5771	KCNMA1	NQO1	PPP5C	REEP5	SCARB2	SYT1	VMA21	
ARL8B	CCT6A	EEF1A1	GNA11	KDELC1	NRAS	PRAF2	RHEB	SCCPDH	SYTL4	VMN2R66	
ARPC1B	CCT7	EEF1A2	GNA13	KIAA0391	NSDHL	PRDX1	RHOA	SCFD1	TAGLN2	VPS16	
ARPC2	CCT8	EEF1B	GNAI2	KIF5B	NSF	PRDX2	RHOC	SDHA	TARS	VPS26A	
ARPC4	CD47	EEF1D	GNAO1	KLC4	NSFL1C	PRDX3	RNF181	SEC22B	TBCA	VPS35	
ARPC5	CDC42	EEF1G	GNB1	KLK8	NUDC	PRDX4	RPAP1	SEC61A2	TCP1	VPS4A	
ASL	CDK1	EEF2	GNB2	KPNB1	OGDH	PRDX6	RPL10A	SEC61B	TECR	WDR1	
ASNA1	CERS5	EIF1AX	GOT2	KRT90	OLA1	PREP	RPL11	SEC63	TFRC	WDR64	
ASNS	CFL1	EIF2S1	GPI	L1CAM	OXCT1	PRKACA	RPL12	SEPT11	TIMM50	XPNPEP1	
ASS1	CHCHD2	EIF2S3X	GRK6	LAMP1	OXNAD1	PRKAR1A	RPL13	SEPT2	TKT	YARS	

dCAD PROTRUSIONS: "IN 2" (139)		
ACLY	NAP1L1	VIM
ACTB	P4HB	YWHAH
ACTG1	PDIA3	YWHAZ
ACTN1	PDIA6	
ACTR1A	PFN1	
ALB	PGAM1	
ALDOA	PHB	
ANXA2	PHB2	
ANXA5	PHGDH	
APRT	PKM	
ARHGDIA	PPIA	
ARPC4	PRDX1	
ASL	PRDX2	
ATP5A1	PRDX4	
ATP5B	PSAT1	
ATP5O	PSMA5	
C1QBP	PSMA6	
CALM1	PSMA7	
CAND1	PSMB1	
CANX	PSMB2	
CAPZA1	PSMD12	
CCT2	RAB10	
CCT3	RAB14	
CCT5	RAB7A	
CCT6A	RAN	
CCT8	RPL12	
CFL1	RPLP0	
CLTC	RPLP2	
CORO1C	RPN2	
CS	RPS12	
CYB5R3	RPS16	
DPYSL2	RPS17	
DSP	RPS23	
EEF1A1	RPS25	
EEF2	RPS28	
EIF3F	RPS3	
ENO1	RPS8	
FASN	RPS9	
FLNB	RPSA	
FN1	RTN4	
FSCN1	SARS	
GAPDH	SCARB2	
GDI1	SDHA	
GOT2	SHMT2	
GSN	SLC1A5	
GSTP1	SLC25A3	
HINT1	SLC25A4	
HIST1H4A	SLC25A5	
HSD17B10	SLC3A2	
HSP90AA1	TARS	
HSP90AB1	TCP1	
HSP90B1	TPI1	
HSPA5	TRAP1	
HSPA8	TRY10	
HSPA9	TUBA1A	
HSPD1	TUBB2A	
HSPE1	TUBB3	
HYOU1	TUBB4B	
ITGB1	TUBB5	
JUP	UBA1	
KPNB1	UBA52	
LDHA	VAT1	
LGALS1	VAT1L	
MAP1B	VCP	
MDH1	VDAC1	
MDH2	VDAC2	
MSN	VDAC3	
MYL6	VGF	

dCAD PROTRUSIONS: "exclusive" (38)
A0A097PUH2
AFDN
AFM
AHSG
ATPIF1
BRK1
C4B
CCR9
CERS5
DPYSL3
FASTKD2
FLG2
GM11214
GM15013
H3F3C
HGFAC
HSPA1B
ITIH1
ITIH4
KCNMA1
KDELC1
KLK8
KRT90
LAPTM5
LNP
OXNAD1
PRNP
PRR27
PYCRL
RHOC
S100A11
SERPINF1
SNX25
SPRY1
SYT1
TRDMT1
VMA21
WDR64

Figure S4
