# Supplementary figures and images for: A novel Microproteomic Approach Using Laser Capture Microdissection to Study Cellular Protrusions
Source: Int J Mol Sci. 2019 Mar 7;20(5):1172. doi: 10.3390/ijms20051172 (PMC6429397; doi:10.3390/ijms20051172)

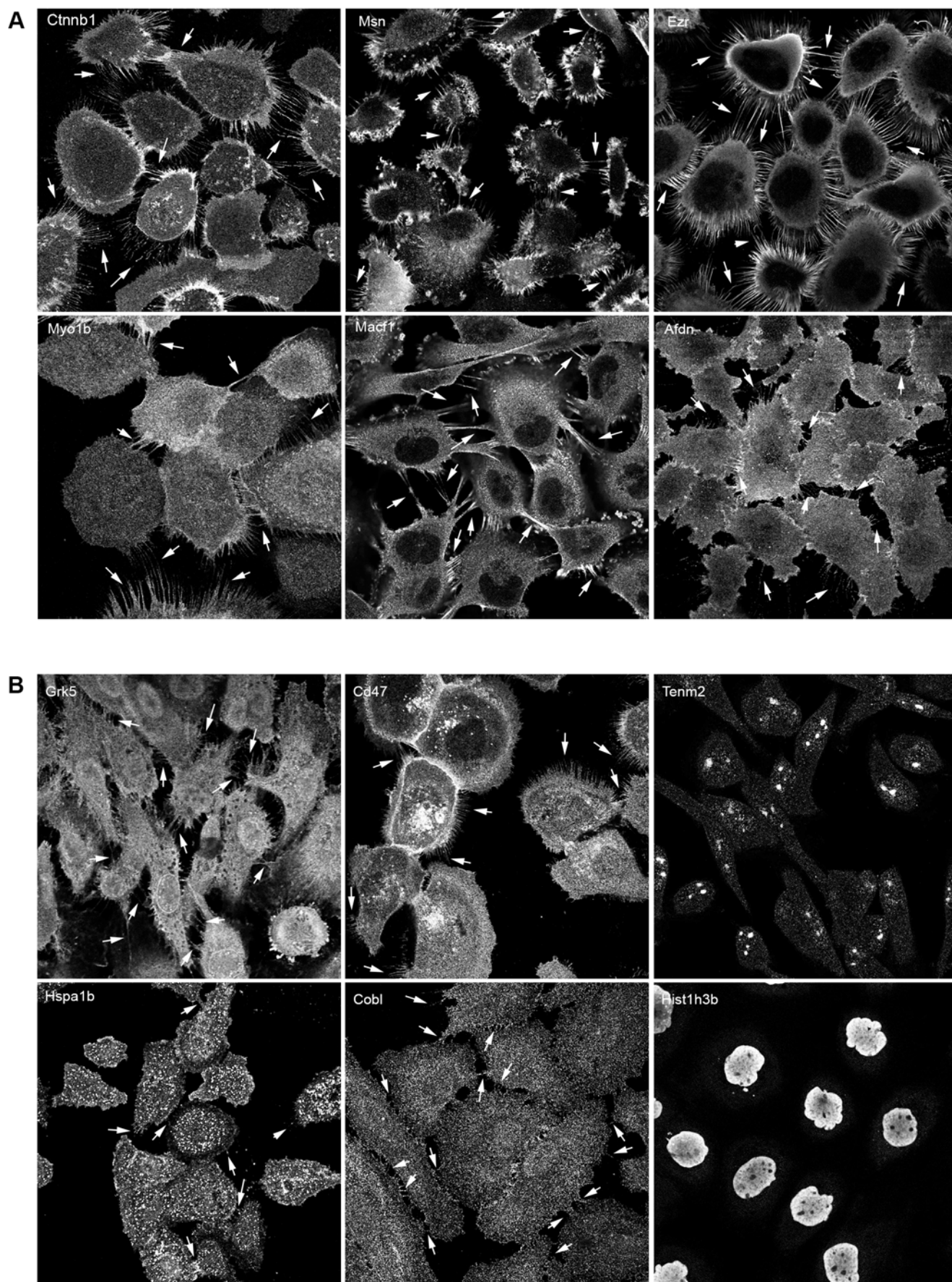

Figure S3

Supplement: Supplementary file 1 [file ijms-20-01172-s001.zip › New-Fig S3-s.pdf]

B

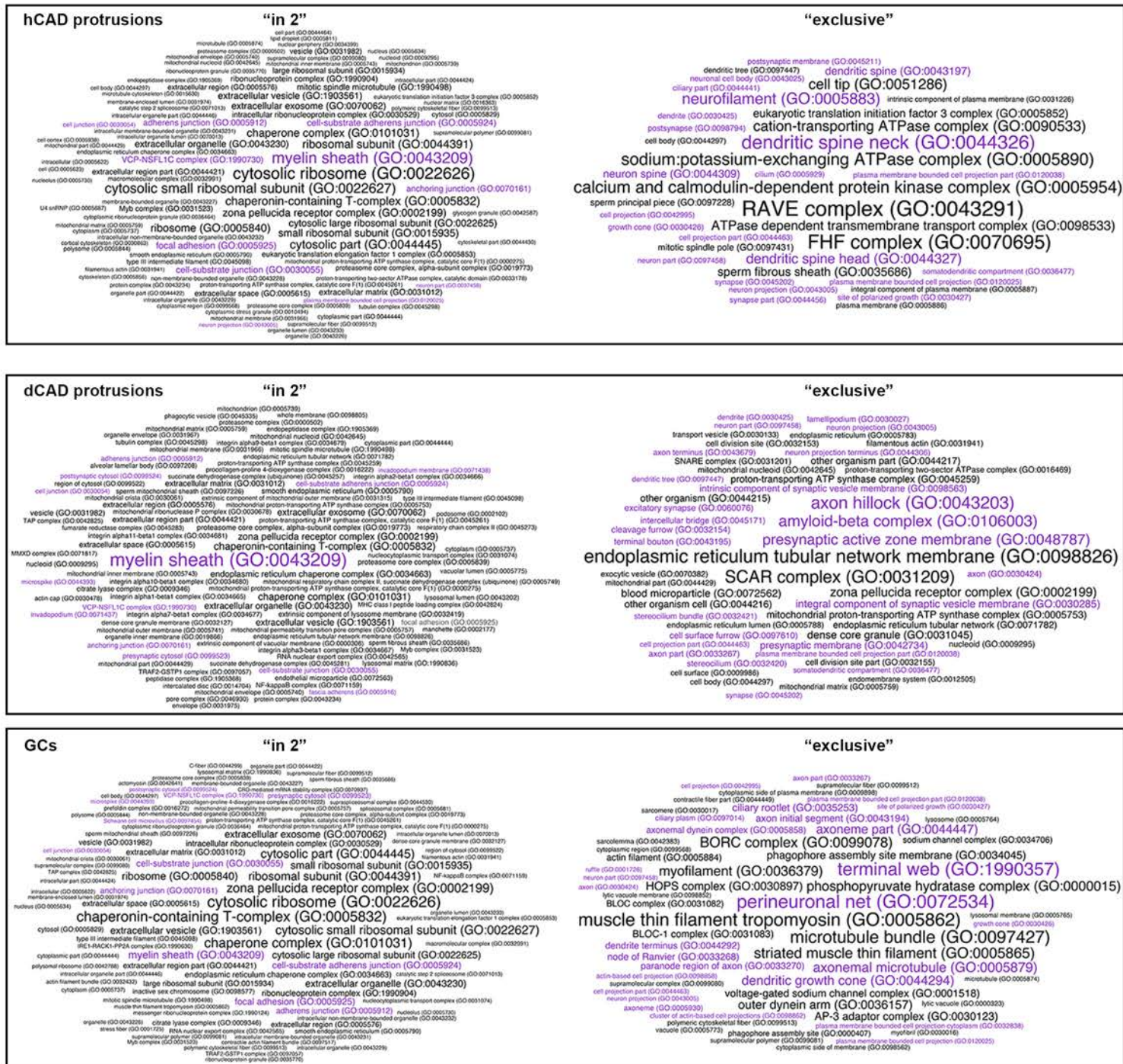

Figure S6

Supplement: Supplementary file 1 [file ijms-20-01172-s001.zip › New-Fig S6B-s.pdf]
