# Supplementary material for: A novel Microproteomic Approach Using Laser Capture Microdissection to Study Cellular Protrusions
Source: Int J Mol Sci. 2019 Mar 7;20(5):1172. doi: 10.3390/ijms20051172 (PMC6429397; doi:10.3390/ijms20051172)
Supplement: Supplementary file 1 [file New-Fig S1-with legend-1-s.pdf › New-Fig S1-with legend-1-s.pdf]

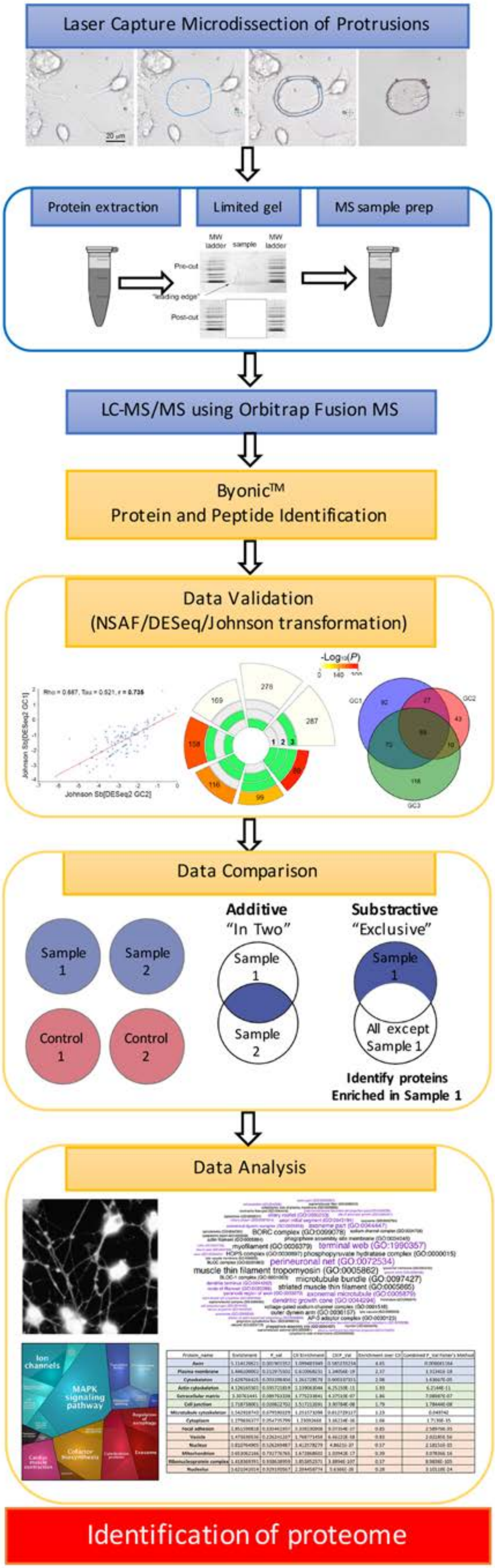

Figure S1: Workflow of the LCM/MS Microproteomic method. Experimental steps (blue) and analysis approaches (orange) of the LCM/MS method are highlighted. (1) LCM is used to isolate specific subtypes of protrusions from fixed samples. (2) After isolation, the collected samples are extracted, run on a limited gel and all of the bands are sent for MS. (3) Samples are prepared and run on an Orbitrap Fusion MS and (4) peptides/proteins are identified using the Byonic software. (5) Data is normalized for peptide length (NSAF), differential expression between samples (DESeq) and normality (Johnson transformation) in order to obtain Pearson correlation values. GC data was compared to published studies for validation of the method. (6) Other subtypes of protrusions were isolated and analyzed using two approaches. An additive method, commonly used in proteomics, which only analyze data obtained in at least two replicate samples, or a subtractive method, where only proteins found exclusively within each subtype of protrusions and not in any other samples are counted as protein hits. (7) Data analysis of the subtypes of protrusions were compared by microscopy (IF/HPA subcellular Database) and using annotations to look at both functions and localization. By subtracting the proteins found in other samples, thereby creating lists of exclusive proteins, we identified lower abundant proteins that are more related to protrusions and (8) demonstrate that subtypes of protrusions have distinct proteomes.
